# Nutrient accumulation and transcriptome patterns during grain development in rice

**DOI:** 10.1093/jxb/erac426

**Published:** 2022-10-22

**Authors:** Zi-Wen Ren, Peter M Kopittke, Fang-Jie Zhao, Peng Wang

**Affiliations:** State Key Laboratory of Crop Genetics and Germplasm Enhancement and Jiangsu Collaborative Innovation Center for Solid Organic Waste Resource Utilization, College of Resources and Environmental Sciences, Nanjing Agricultural University, Nanjing 210095, China; Centre for Agriculture and Health, Academy for Advanced Interdisciplinary Studies, Nanjing Agricultural University, Nanjing 210095, China; The University of Queensland, School of Agriculture and Food Sciences, St Lucia, Queensland, 4072, Australia; State Key Laboratory of Crop Genetics and Germplasm Enhancement and Jiangsu Collaborative Innovation Center for Solid Organic Waste Resource Utilization, College of Resources and Environmental Sciences, Nanjing Agricultural University, Nanjing 210095, China; State Key Laboratory of Crop Genetics and Germplasm Enhancement and Jiangsu Collaborative Innovation Center for Solid Organic Waste Resource Utilization, College of Resources and Environmental Sciences, Nanjing Agricultural University, Nanjing 210095, China; Centre for Agriculture and Health, Academy for Advanced Interdisciplinary Studies, Nanjing Agricultural University, Nanjing 210095, China; Lawrence Berkeley National Laboratory, USA

**Keywords:** Growth curve, mineral elements, redistribution, rice grain filling

## Abstract

Rice is an important source of calories and mineral nutrients for more than half of the world’s population. The accumulation of essential and toxic mineral elements in rice grain affects its nutritional quality and safety. However, the patterns and processes by which different elements progressively accumulate during grain filling remain largely unknown. In the present study, we investigated temporal changes in dry matter, elemental concentrations, and the transcriptome in the grain of field-grown rice. We also investigated the effects of seed setting rate and the position of the grain within the rice panicle on element accumulation. Three different patterns of accumulation were observed: (i) elements including K, Mn, B, and Ca showed an early accumulation pattern; (ii) dry matter and elements including N, P, S, Mg, Cu, Zn, Mo, As, and Cd showed a mid accumulation pattern; and (iii) elements such as Fe showed a gradual increase pattern. These different accumulation patterns can be explained by the differences in the biogeochemical behavior of the various elements in the soil, as well as differences in plant nutrient redistribution, gene expression, and the sink–source relationship. These results improve our knowledge of the dynamics of elemental accumulation in rice grain and are helpful for identification of functional genes mediating the translocation of elements to grain.

## Teaser

Mineral nutrients accumulated in rice grains in three distinct patterns during grain development, with corresponding patterns of gene expression based on transcriptomic analysis. Different nutrients displayed different spatial distribution patterns in the grain.

## Introduction

The accumulation of mineral nutrients in cereal grains has important implications for human health Huang *et al.* (2020). The inadequate intake of micronutrients and vitamins whilst having a satisfactory energy intake is known as ‘hidden hunger’ (Saltzman *et al.*, 2013). For example, the insufficient dietary intake of iron (Fe) and zinc (Zn) affects the nutritional health of nearly 2 billion people globally and is associated with many diseases (Allen *et al.*, 2006; Wessells and Brown, 2012). Rice (*Oryza sativa*) is an important source of both calories and mineral nutrients for half the world’s population, especially in Asia (Yuan, 2014). Increasing the contents of essential nutrients, especially Fe and Zn, in the edible portions of rice and other staple crops through biofortification is considered to be an economical and effective way to address ‘hidden hunger’ (White and Broadley, 2009). Therefore, understanding the accumulation patterns of both dry matter and mineral nutrients and the mobility of nutrients within rice grains during the filling stage is important for improving nutritional quality.

Rice grains have three developmental stages: (i) embryo morphogenesis [~0–10 days after fertilization (DAF)]; (ii) endosperm filling (~10–30 DAF); and (iii) seed maturation (30 DAF to maturation) (An *et al.*, 2020). During the first stage, the embryo undergoes the globular stage until 3 DAF. Starting from 5 DAF, the first leaf primordium is recognizable. Subsequently, endosperm filling commences, with compounds including protein bodies being stored, causing the aleurone and starchy endosperm to form during this stage. Most morphogenetic events are completed by 10 DAF (Krishnan and Dayanandan, 2003; Itoh *et al.*, 2005; Zhu *et al.*, 2011; Fu *et al.*, 2013). Grain filling reaches the maximum rate between 10 and 20 DAF (Zhu *et al.*, 2011; Fu *et al.*, 2013). After 21 DAF, starch granules are formed in the starchy endosperm and the endosperm enters the maturation stage (Wu *et al.*, 2016a), when the embryo and endosperm continue to dehydrate until maturity (Wu *et al.*, 2016b). Previous studies have examined changes in dry matter and water content during rice grain development (An *et al.*, 2020; Tao *et al.*, 2022). However, the patterns of nutrient accumulation within the grain are unclear (An *et al.*, 2020).

Of interest in the present study is the accumulation of mineral nutrients and toxic elements in the grain, a process that is influenced by multiple factors. First, uptake and accumulation of nutrients depend on the availability of nutrients in the paddy soil. For example, available copper (Cu), manganese (Mn), and Fe in soil decreases with increasing pH (Rengel, 2015; Antoniadis *et al.*, 2017; Dede *et al.*, 2017). In addition, many studies have shown that intermittent flooding and drainage of the paddy soil during rice growth alters the availability of both arsenic (As) and cadmium (Cd). Both Cd and As are elements sensitive to soil redox. The availability of Cd decreases when the paddy is flooded but increases markedly once drained. In contrast, the availability of As is highest when the paddy is flooded and lowest when drained (Zhao and Wang, 2020). It has been shown that the majority (>90%) of the Cd in the grain is derived from root uptake from the soil during grain filling (when the paddy is drained) but, in contrast, As accumulates in the grain due to remobilization from the vegetative tissues during grain filling (Arao *et al.*, 2009; Li *et al.*, 2009; Ma *et al.*, 2014; B.Y. Huang *et al.*, 2021). Unlike these two toxic elements, it is unclear whether the accumulation of essential elements in the grain is also affected by changes in elemental availability in the paddy soil caused by drainage prior to harvest.

Second, the accumulation of elements in the grain is influenced by the internal supply of elements from vegetative tissues to the grain (Martin and Marschner, 1988). In wheat (*Triticum aestivum*), the redistribution of nitrogen (N) from the leaf to the grain accounts for ~40% of the N in the grain, whilst redistribution from the glume accounts for 23% of the grain N, the stem for 23%, and the root for 16% (Simpson *et al.*, 1983). In rice, the contribution of the leaf to grain N has been reported to be 24%, and that of glume to grain N is 9.8–10% (Zhang *et al.*, 2017). Concentrations of phosphorus (P) in leaves and stems tend to decrease during the filling stage, contributing 20% of the grain P (Rose *et al.*, 2010; Julia *et al.*, 2016). Similarly, nearly 60% of the grain potassium (K) is derived from vegetative organs (Li *et al.*, 2014; Sun *et al.*, 2016). Several studies have demonstrated the importance of redistribution of nutrients from vegetative organs to seeds (Sperotto *et al.*, 2012; Wei *et al.*, 2012; Jiang-Xing *et al.*, 2013; Sperotto, 2013; Punshon *et al.*, 2018). For example, flag leaf exposed to As during grain developing caused higher grain As concentrations (Punshon *et al.*, 2018). Grain Fe and Zn accumulation are also redistributed from other leaves or the stem/sheath when there is a lack of flag leaves (Sperotto, 2013). The redistribution of other mineral elements [such as calcium (Ca), magnesium (Mg), and molybdenum (Mo)] from vegetative organs to rice grain under field conditions is not well understood.

Third, the accumulation of elements in the grain involves multiple steps of cross-membrane transport mediated by various transporters. Membrane transporters are required for the loading of nutrients into the developing rice grain, including efflux transporters on the maternal tissue side and influx transporters on the filial side. For example, *OsYSL15* and *OsYSL18*, expressed in developing grains, are likely to be involved in grain Fe(III)-deoxymugineic acid [Fe(III)-DMA] loading (Aoyama *et al.*, 2009; Lee *et al.*, 2009). Furthermore, OsYSL9 is a membrane transporter mediating the internal transport of Fe(II)–nicotianamine complex [Fe(II)–NA] and Fe(III)–DMA, especially from the endosperm to the embryo in developing seeds (Senoura *et al.*, 2017). OsVIT2 is important for aleurone Fe accumulation via sequestering Fe into vacuoles in the aleurone layer (Che *et al.*, 2021). Transcription of these genes may be dependent on the developmental stage (Wu *et al.*, 2020; Tao *et al.*, 2022), thus affecting the accumulation and distribution of elements within the grain. However, whether the transcriptional dynamics of these genes affect nutrient accumulation in grains remains to be investigated.

Finally, the spatial distribution of mineral elements within the grain is likely to be heterogenous, a feature that can affect mineral concentrations and bioavailability to humans after brown rice is polished. In this regard, microanalyses in the grains of wheat and rice have shown that P, K, Ca, Mn, Fe, and Zn are often distributed in a similar manner, with the highest concentrations being observed in the aleurone and the embryo (especially the scutellum), and the lowest concentrations in the endosperm (Mazzolini *et al.*, 1985; Heard *et al.*, 2002; Gu *et al.*, 2020). Furthermore, it has been shown that P, K, Mg, Ca, and Fe are strongly co-localized with phytate in the aleurone layer (Heard *et al.*, 2002; Moore *et al.*, 2012). This distribution within the grain explains why the concentrations of many mineral elements are low in milled (i.e. polished), de-embryonated grains. The mechanisms that lead to the differences in the spatial distribution of these elements within the grain remain unclear.

The aim of the present study was to examine temporal changes in element concentrations in the grain of rice. This temporal dynamics affect the spatial distribution of different elements within the grain. We examined dynamic changes in the bulk concentrations of elements in the grain of field-grown rice and related this information to transcriptomics in the grain from RNA-sequencing. In addition, we used synchrotron-based micro-focused X-ray fluorescence (μ-XRF) for *in situ* analyses of the distribution of elements within the various tissues of the grain. This study identified three patterns of element accumulation, which are associated with gene expression patterns during the filling stage. These results improve our knowledge of the accumulation dynamics of elements in rice grain and provide important information for identification of functional genes mediating the translocation or loading of elements into the grain.

## Materials and methods

### Plant materials and growth conditions

Rice (cv. Nangen 9108, *Oryza sativa* L. ssp. *japonica*) was grown to maturity in an uncontaminated paddy field in Nanjing, China, from June to September 2020. The paddy was flooded during plant growth, except for periods during the late tillering stage and the mid to late grain-filling stage when the paddy water was drained. Basic soil properties are shown in Supplementary Table S1. A plot of 10 m^2^ was divided in the center as the sampling area. The main panicles were marked at the time of flowering. The date of flowering was recorded for individual spikelets on the main panicles. The developing grain on the marked panicles were collected at 5, 9, 13, 17, 21, 25, and 30 DAF, and ~60 of the whole panicles were randomly collected at each period from the 10 m^2^ plot. The marked spikelets (~180 grains in total) were collected from the 60 panicles. The spikelets were divided into six aliquots (~30 grains from 10 panicles as one replicate). The grains from three replicates were de-husked and immediately frozen in liquid nitrogen before being stored at –80 °C for transcriptomic and quantitative real-time PCR (qRT-PCR) analysis. Developing grain, node I, and the flag leaf on the marked panicles were collected at 5, 9, 13, 17, 21, 25, and 30 DAF, and each tissue had four biological replicates from four individual rice plants randomly located in the 10 m^2^ plot. The plant tissues were dried at 65 °C to a constant mass for elemental determination. Developing grains were photographed using a SZX2 Zoom Stereo Microscope (Olympus, Japan).

To investigate whether there is spatial variation in the accumulation of elements within the different positions of a panicle, mature marked main panicles were collected and divided into seven parts (apical to basal, A–G). Grains from each primary rachis branch were pooled as a sample, de-husked, and dried for analysis. Three main panicles from three individual rice plants which were randomly arranged in the plot were collected as three replicates. In order to investigate whether elemental accumulation within the grain was related to seed setting rate, we manually pruned spikelets at the anthesis (R4 stage) to control the seed setting rate per main panicle (Counce *et al.*, 2000; Sperotto *et al.*, 2013). The main panicles of three plants with uniform growth and heading and flowering on the same day were selected for pruning spikelets. A 100% seed setting rate referred to the control group which did not receive any treatment; a 75% seed setting rate referred to the treatment which removed a quarter of the primary rachis branches from basal to apical; and a 50% seed setting rate referred to the treatment which removed half of the primary rachis branches. Rice grains in the apical spikelets of the main panicle were harvested at full maturity (R9 stage), oven-dried, and de-husked for elemental analysis (Counce *et al.*, 2000; Sperotto *et al.*, 2013). Four spikelets from four individual rice plants were collected as four replicates.

### Library preparations for RNA-seq

Approximately 15 freeze-dried grain samples from five temporal grain developmental stages (5–21 DAF) were used for transcriptomics analysis, with each stage having three biological replicates. Total RNA from grain samples was isolated with TRIzol reagent, according to the manufacturer’s instructions (Invitrogen, Carlsbad, CA, USA). RNA quality was assessed by an Agilent 2100 Bioanalyzer (Agilent Technologies, Palo Alto, CA, USA) and checked using RNase-free agarose gel electrophoresis. After total RNA was extracted, eukaryotic mRNA was enriched by oligo(dT) beads, while prokaryotic mRNA was enriched by removing rRNA using the Ribo-Zero™ Magnetic Kit (Epicentre, Madison, WI, USA). The enriched mRNA was fragmented into short fragments using fragmentation buffer and reverse transcribed into cDNA with random primers. Second-strand cDNA was synthesized by DNA polymerase I, RNase H, dNTP, and buffer. Then the cDNA fragments were purified with a QiaQuick PCR extraction kit (Qiagen, Venlo, The Netherlands), end-repaired, poly(A) added, and ligated to Illumina sequencing adaptors. cDNA libraries were sequenced on an Illumina HiSeq2500 sequencing platform by Gene Denovo Biotechnology Co. (Guangzhou, China) to generate 150 bp paired-end reads. Raw sequence data have been deposited in the NCBI SRA database, with accession numbers SRR17706479, SRR17709283, SRR17709378, SRR17709379, and SRR17709726.

### RNA-seq data processing and trend analysis

The raw reads were filtered using fastp (version 0.18.0) to remove adaptor sequences and low-quality reads, including >10% of unknown nucleotides and low-quality reads containing >50% of low-quality (Q value ≤20) bases (Chen *et al.*, 2018). Data filtering of reads is shown in Supplementary Table S2. The number of clean reads in each sample of this study was >36 000 000, accounting for ~98% of the total reads. The number of high-quality bases in each sample was >5 500 000 000, accounting for ~98% of the total sequenced bases, and >97% of the reads were compared with the reference genome (Supplementary Table S2). Paired-end clean reads were mapped to the rice *japonica* reference genome (cv. Nipponbare reference genome IRGSP1.0, http://rice.plantbiology.msu.edu) using HISAT2. 2.4 with ‘rna-strandness RF’ and other parameters set as defaults (Kim *et al.*, 2015), followed by gene mapping to the Gene Ontology (GO) and Kyoto Encyclopedia of Genes and Genomes (KEGG) databases to annotate their potential biological processes and metabolic pathways (Ashburner *et al.*, 2000).

Fragment per kilobase of transcript per million mapped reads (FPKM) was calculated to normalize the original gene expression level. These FPKM data were used to reveal the relationship among samples by principal component analysis (PCA). Subsequently, the temporal expression profile of genes was analyzed based on the FPKM values, and Short Time-series Expression Miner (STEM) software (http://www.cs.cmu.edu/~jernst/stem) was used to display the 20 temporal expression profiles during the five sampling stages (profiles 20, log2ratio ≥1). All samples are compared with the first sample, with the expression fold calculated. Once the temporal expression profile of genes was analyzed, KEGG enrichment analyses were performed for each profile to find the biological function of the profile, with the pathways with *P* ≤0.05 being defined as the significantly changed KEGG pathways. The above statistical analyses were performed by the OmicShare tools (https://www.omicsmart.com).

### qRT-PCR analysis of the candidate genes

Relative expression of the genes of interest was confirmed by qRT-PCR. The developing grains (5–21 DAF) used in this experiment were the same as used for RNA-seq, which were collected in the same growth season from different individual plants. The developing grains (5–21 DAF) were sampled for the extraction of total RNA with a Plant Total RNA Extraction Kit (BioTeke, Beijing, China). From the samples, 1 μg of total RNA was converted to cDNA with the HiScript II 1st Strand cDNA Synthesis Kit (Vazyme, Nanjing, China). qRT-PCR was performed with SYBR Green Master Mix (Vazyme, Nanjing, China) on a Real-Time PCR Detection Systems (CFX96, Bio-Rad). *Actin* and *HistoneH3* were used as an internal control for gene expression in grain at the grain-filling stage. Relative expression levels were calculated by the comparative Ct method (Livak and Schmittgen, 2001; Che *et al.*, 2021). Three independent biological replicates were performed for each treatment. The primer sequences are presented in Supplementary Table S3.

### Determination of element concentrations in rice tissues

Grain samples (~ 250 mg) were digested in high-purity concentrated HNO_3_ in a microwave digestion system (CEM, 149 Mars, USA). Dried vegetative tissues (~250 mg) were ground to a fine powder and digested in 5 ml of HClO_4_/HNO_3_ mixed acids (15/85, v/v) in a heating block (Zhao *et al.*, 1994), with the digestion program presented in Supplementary Table S4. Elemental concentrations in the digests were determined using inductively coupled plasma MS (ICP-MS; Perkin Elmer NexION300X, USA). Quality control included the addition of indium (20 μg l^–1^ in 2% HNO_3_) as the internal standard and the use of procedural blanks, duplicates, and repeated analysis of rice-certified reference (GBW10045a for rice grain and GBW10015a for plant).

For tissue N analysis, ~50 mg of the grain powder was digested by 5 ml of 98% H_2_SO_4_ and 1 ml of 30% H_2_O_2_ at 270 °C for 1 h (Cai *et al.*, 2011). The digest was diluted to 100 ml with distilled water after cooling. The N concentration was determined by colorimetric continuous flow analysis (Auto Analyser 3, Bran + Luebbe, Germany).

In the present study, C_conc._ refers to the concentration of an element of a certain tissue (mg kg^–1^ or μg kg^–1^), while C_cont._ refers to the quantity of an element (mg or μg) in the whole tissue.

### Logistic growth model

The temporal patterns of elemental accumulation in grain can be described using the logistic equation (Equation 1), and the accumulation rate was derived from the first derivative of the equation (Blumberg, 1968):


$$\begin{array}{l} \;W\;=\;a/[1+\;e\hat{\;}\left(-k\left(x-x_{c}\right)\right] \end{array}$$ (1)

where *W* is dry matter or elemental content per grain (mg, μg, or pg) at a certain DAF (*x*); *x*_c_ is the DAF at the inflection point, and *a* is the maximum of content during the grain-filling stage; *k* is the exponential growth rate (i.e. increase in content per unit time). The elemental accumulation rate was calculated from the first derivative of this equation.

### Calculation of net accumulation and contribution ratio

To estimate the apparent redistribution from the flag leaf and node I to the grain, their net accumulation (Equation 2) and contribution ratio of elements (Equation 3) were calculated as:


$$\begin{array}{l} \Delta C_{i}=\;C_{30}^{i}-C_{5}^{i} \end{array}$$ (2)

$$\begin{array}{l} R_{i}\;=\;\left|\;\Delta \;C_{i}\right|/C_{30}^{grain}\;\times 100\% \end{array}$$ (3)

where *i* represents the grain, node I, or flag leaf; $C_{5}^{i}$ and $C_{30}^{i}$ are the element content at 5 or 30 DAF (mg, μg, or pg); $\Delta C_{i}\;$ is the net accumulation of an element (mg, μg, or pg); $C_{30}^{grain}$ is the grain element content (mg, μg, or pg) at 30 DAF; and $R_{i}$ is the contribution ratio of the element (%).

### Soil incubation experiment

To investigate the temporal changes in soluble element concentration in paddy soil during grain filling, a soil incubation experiment was conducted to simulate the occurrence of flooded and drained management in the paddy field. In brief, 1000 g of soil (<2 mm) was placed in a 1000 ml plastic bottle and 500 ml of deionized water were added to form a 5 cm layer of standing water above the soil surface. This soil incubation was replicated in four bottles at 28 °C in darkness. During the soil flooding phase (0–15 d), a standing layer of water (~5 cm) was maintained in plastic bottles, while during the soil oxidation phase (15–30 d), water was slowly drained through holes (diameter=5 mm) at the bottom of the bottles and the soils were then maintained under moist but aerobic conditions. Soil porewater was collected every 5 d using a porewater sampler (~10 cm underneath the soil surface, Rhizon Soil Moisture Samplers, The Netherlands) and pH was determined immediately using a pH electrode that was inserted from the top to the center of the soil layer. Soil redox potential (Eh) was determined at 10 cm underneath the soil surface using a combination Pt electrode. The porewater samples were acidified with concentrated HCl (100:1; v:v) and filtered through 0.22 μm membrane filters before elemental concentration analysis by ICP-MS (Zhao *et al.*, 2013).

### Elemental distribution using μ-XRF

Grains were embedded in an epoxy resin (Araldite GY 191, Huntsman, Australia) and cut into 100 μm thick transverse sections. The sections were placed between two pieces of 4 μm thick Ultralene film and analyzed by μ-XRF at the XFM beamline at the Australian Synchrotron (Australia), where an in-vacuum undulator is used to produce a brilliant X-ray beam. The step size (virtual pixel size) was 8 µm×8 µm with a dwell time of 80 ms per pixel. The incident energy was 18.5 keV with a photon flux of 8.4 × 10^9^ photons s^–1^. The X-ray fluorescence emitted by the specimen was collected using the 384 element Maia detector in a backscatter geometry. The XRF spectra were analyzed using GeoPIXE, and images with quantitative data were generated using the dynamic analysis method (Gu *et al.*, 2020).

### Statistical analysis

All data are presented as the mean ±SE. ANOVA was used to test the significance of different filling stage effects, followed by comparisons of treatment means using Tukey’s test (*P*<0.05). Statistical analyses were performed using IBM SPSS Statistics v. 25.

## Results

### Pattern of grain dry matter accumulation

**Fig. 1.**
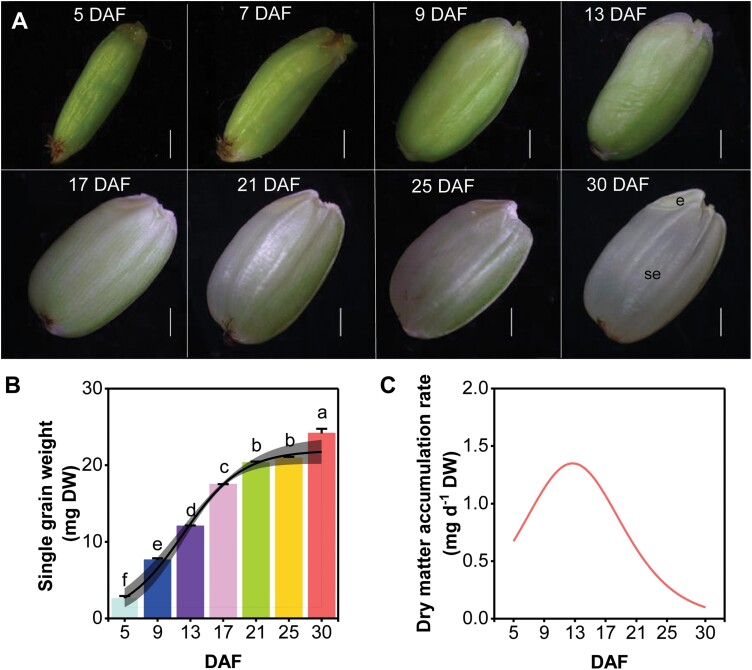
Dry matter accumulation in grains and seed morphology after fertilization. (A) Grain morphology from grain filling to maturity. (B) Increase in single grain weight from the grain-filling stage to maturity. (C) Dry matter accumulation rate. Data at different developmental stages were obtained from three biological replicates; each biological replicate consisted of 10–80 grains collected from marked panicles. Different letters indicate significant differences among different developmental stages (Tukey’s test, *P*<0.05). The weight of three individual grain was used to derive accumulation rates during different development periods (5–30 DAF). Dark gray (#808080) is the 95% confidence interval, which was calculated using *t*-distributions based on sample variance and population mean. DAF, days after fertilization; e, embryo; se, starchy endosperm. Scale bars=1 mm.

During the milk ripening stage (5–21 DAF), the dry mass of grains increased rapidly and white rice pulp appeared inside the green grain (Fig. 1A), with the embryo visually observed at 17 DAF. Between 17 and 25 DAF, grains gradually hardened, and the green coloring of the dorsal longitudinal furrows gradually faded, indicating that the grain was entering the waxy maturity stage (Fig. 1A). During this stage, the grain surface color changed from green to milky white and the embryo was fully formed by 30 DAF (Fig. 1A). The DW of the grain increased steadily from 5 to 30 DAF (Fig. 1B), with the rate increasing from 0.67 mg d^–1^ DW at 5 DAF to a peak value of 1.35 mg d^–1^ DW at 13 DAF and then gradually decreasing to 0.10 mg d^–1^ DW at 30 DAF (Fig. 1C).

### Patterns of element accumulation

The contents of all elements in the grain, except Ca and K, increased between 5 and 30 DAF (Supplementary Fig. S1). For Ca and K, an interesting trend was observed whereby their contents remained essentially constant from 10 to 30 DAF (Supplementary Fig. S1). In marked contrast to Ca and K, the Fe content in the grain continued to increase throughout grain filling.

While the total contents of elements increased, the concentrations of 12 elements (N, P, S, K, Ca, Mg, Mn, Zn, B, Mo, As, and Cd) decreased between 5 and 30 DAF (Supplementary Fig. S2). The magnitude of the decrease was in the order Ca>Mn>B>K>Cd>Zn>N>As>S>P>Mo>Mg (Supplementary Fig. S2). In particular, the concentrations of K, Ca, Mn, and B decreased sharply from 5 to 9–13 DAF (Supplementary Fig. S2D, E, G, K). The concentrations of Ca and Mn decreased by ~60–68% between 5 and 9 DAF (Supplementary Fig. S2E ,G), whereas the concentrations of K and B decreased more steadily over time (Supplementary Fig. S2D, K). In contrast to these elements, the concentration of Cu in grain remained comparatively stable during the entire period (Supplementary Fig. S2I). The pattern of change in the Fe concentration differed from that of the other elements examined, showing an initial decrease of ~60% between 5 and 9 DAF and an increase of 84% between 21 and 30 DAF (Supplementary Fig. S2H).

**Fig. 2.**
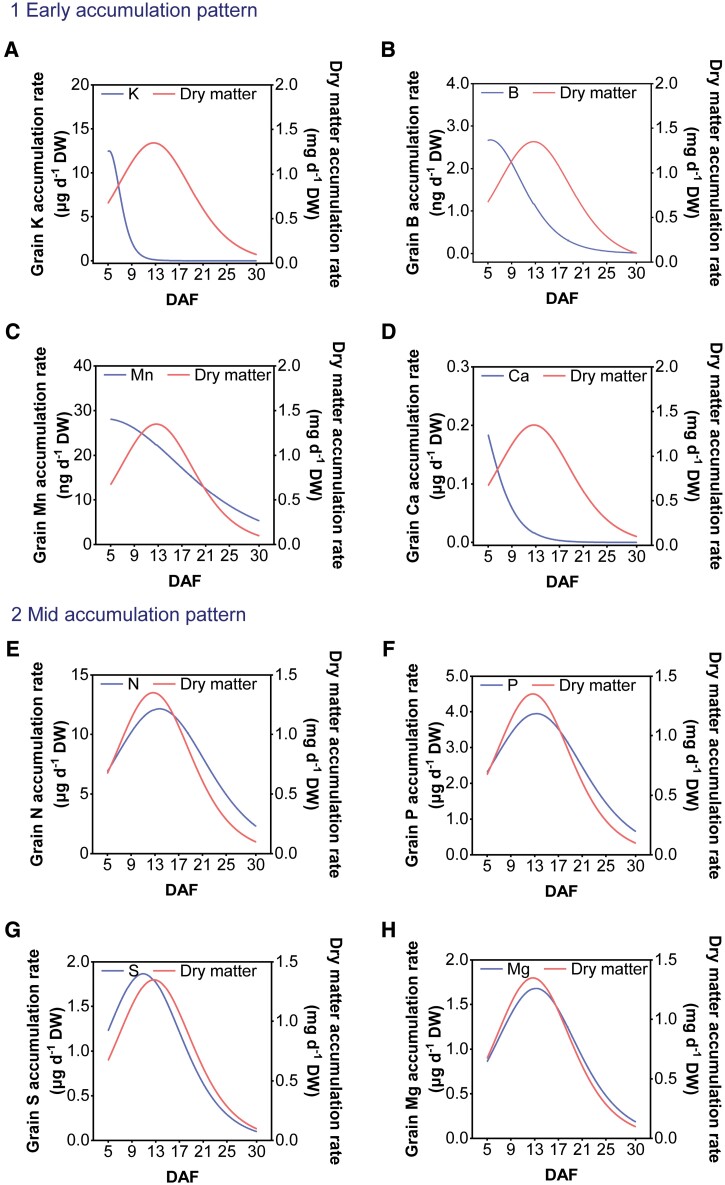

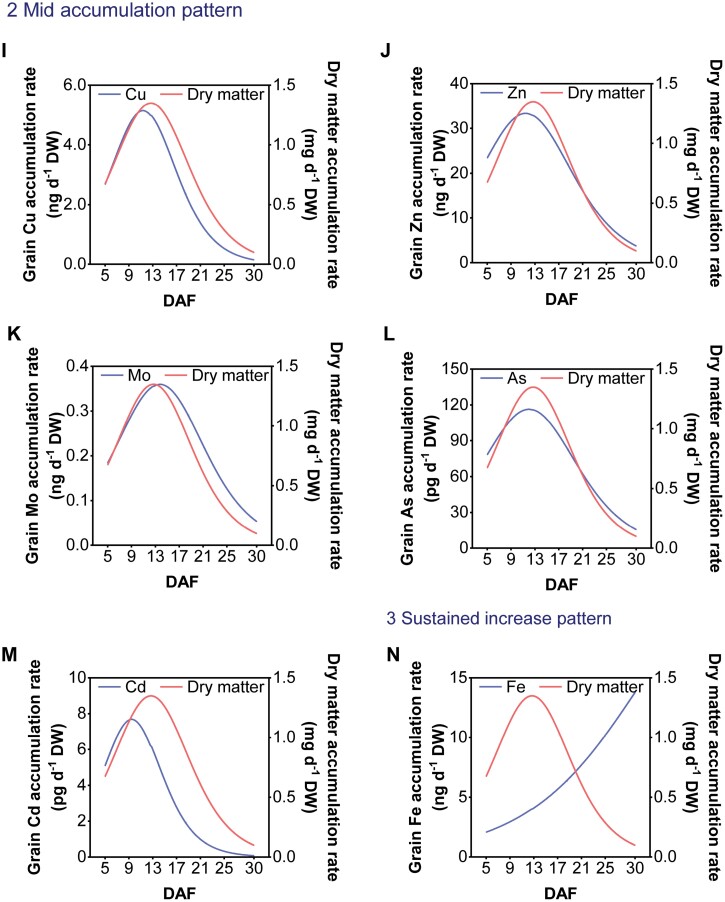
Element accumulation rate (μg d^–1^, ng d^–1^, or pg d^–1^ DW). Early accumulation pattern (A–D). Mid accumulation pattern (E–M). Sustained increase pattern (N). DAF, days after fertilization. The weight of four individual grain was used to derive accumulation rates at different development periods (5–30 DAF). Red (#ff6666) and royal blue (#5e72ff) lines within graphs indicate dry matter and element accumulation rate, respectively.

The rates of element accumulation can be estimated from the changes in their contents and in dry matter accumulation. Three patterns of elemental accumulation were identified (Fig. 2). The first pattern, the ‘early accumulation pattern’, was observed for K, B, Mn, and Ca. These four elements were loaded rapidly into the grain during the early phase of grain development (5 DAF, i.e. prior to the peak of dry matter) and then their accumulation rates decreased to a low level, with the rate of decrease in Mn and B being slower than for both K and Ca (Fig. 2A–D). Although the rate of Mn accumulation decreased consistently over time, the rate of accumulation remained at 5.32 ng d^–1^ DW at 30 DAF, indicating that Mn continued to accumulate through to the ripening stage (Fig. 2C). The second pattern, the ‘mid accumulation pattern’, was observed for four macronutrients (N, P, S, and Mg) (Fig. 2E–H), three micronutrients (Cu, Zn, and Mo) (Fig. 2I–K), and two non-essential elements (As and Cd) (Fig. 2L, M). Specifically, changes in the accumulation rate patterns of N, P, and Mg were almost identical to that of dry matter, with the maximum accumulation rates being 12.1 μg d^–1^ for N, 3.9 μg d^–1^ for P, and 1.6 μg d^–1^ for Mg (Fig. 2E, F, H). For S, Cu, Zn, As, and Cd, the maximum accumulation rates (1.8 μg d^–1^ for S, 5.1 ng d^–1^ for Cu, 33.3 ng d^–1^ for Zn, 116.3 pg d^–1^ for As, and 7.6 pg d^–1^ for Cd) were attained slightly earlier than for dry matter (Fig. 2G, I, J, L, M), whilst Mo reached its maximum accumulation rates (0.3 ng d^–1^) at 14 DAF which was slightly later than for dry matter (Fig. 2K). The third pattern, the ‘sustained increase pattern’, was observed for Fe only. Fe reached its maximum accumulation rate (13.8 ng d^–1^) at 30 DAF, which was considerably later than for dry matter (Fig. 2N).

### Gene expression patterns during grain-filling stages

**Fig. 3.**
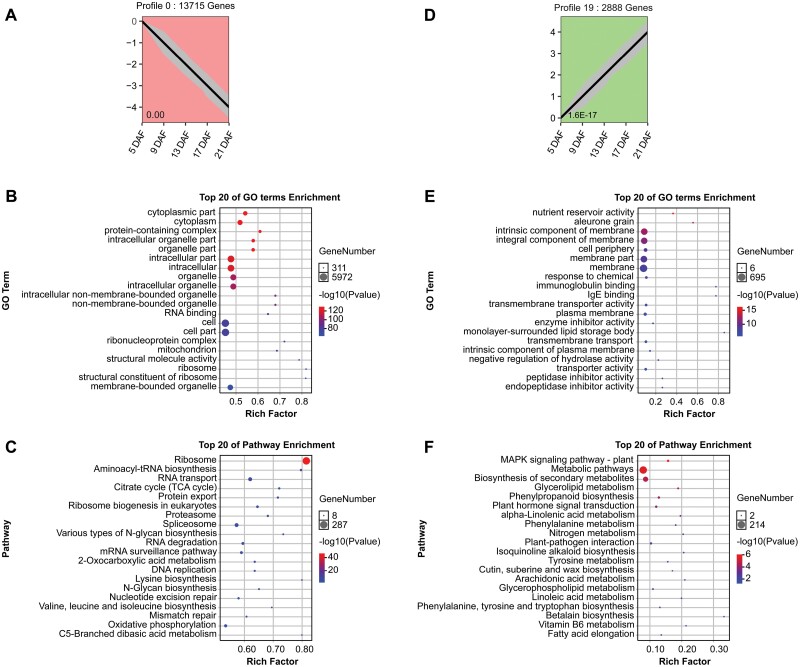

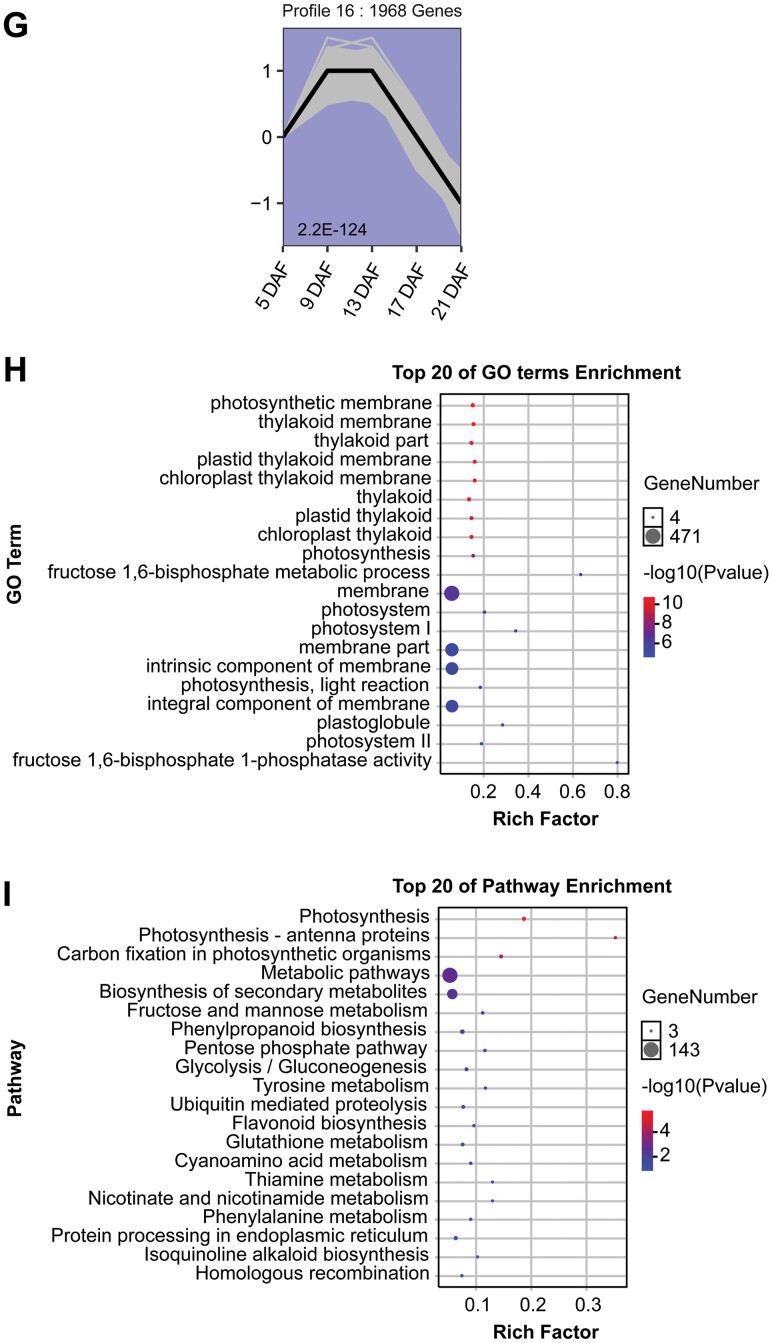
Temporal dynamics of gene expression in developing grain (5–21 DAF). (A) Pattern of early response genes in developing grain. (B–C) GO function and KEGG enrichment analysis of the early response pattern. (D) Pattern of sustained increase genes in developing grain. (E and F) GO function and KEGG enrichment analysis of the sustained increase pattern. (G) Pattern of middle response genes in developing grain. (H and I) GO function and KEGG enrichment analysis of the middle response pattern. *P*-values are represented below each profile, which represent the significance of the genes concentrated in that profile. Each gray line represents a gene, and the bold black line represents dynamic expression patterns. Colors indicate three gene expression trends: red (#fe9999), early response pattern; green (#99fe99), sustained increase pattern; blue (#9999fe), middle response pattern. DAF, days after fertilization.

To relate gene expression to the accumulation rates of dry matter and elements in the grain, we analyzed the transcriptomes of developing grain at different stages (5–21 DAF). RNA-seq of 15 cDNA libraries from the five grain developmental stages generated ~604 million clean reads (Supplementary Table S2). Based on gene expression, PCA was used to estimate the distance relationship among the 15 samples (Supplementary Fig. S3). PCA score plots showed a clear separation between samples from different grain developmental stages, forming five groups. The five developmental stages were separated mainly along PC1 (explaining 84.4% of the variations) with a left-to-right trend. Expression profiles of the whole gene set showed that 27 992 genes could be classified into 20 different expression patterns (Supplementary Fig. S4). Of these, three were identified as representing a significant (*P*<0.05) temporal pattern (i.e. profile 0, profile 19, and profile 16) (Supplementary Fig. S4). These three profiles correspond to the patterns of element accumulation (Fig. 2), with profiles 0, 16, and 19 representing the early response, middle response, and sustained increase patterns, respectively. For each of the three profiles, the 20 most over-represented GO terms and KEGG pathways are shown in Fig. 3 to highlight the biological functions of the genes.

Profile 0 included 13 715 genes, whose expression decreased with developmental stage (Fig. 3A). Genes in profile 0 are enriched in the GO categories including morphogenesis-related processes such as cytoplasmic part, organelle part, membrane part, and ribosome part (Fig. 3B), while the enriched KEGG pathways are associated with translation, protein biosynthesis, providing energy, DNA replication, and metabolic processes (Fig. 3C). Multiple ribosomal proteins (RPs) were found to have significantly higher transcript abundance at 5 DAF, suggesting that morphogenesis is active at this early stage (Supplementary Fig. S5). In contrast, the 2888 genes from profile 19 presented an opposite trend, with low expression at 5 DAF and increasing expression towards 21 DAF. These genes are mainly enriched in the pathways of secondary metabolism/biosynthesis and signal transduction (Fig. 3D–F). The middle response profile 16 included 2888 genes, which are enriched in the KEGG pathways associated with photosynthesis and metabolic processes, and the related proteins are mainly located in the membranes (Fig. 3H, I).

**Fig. 4.**
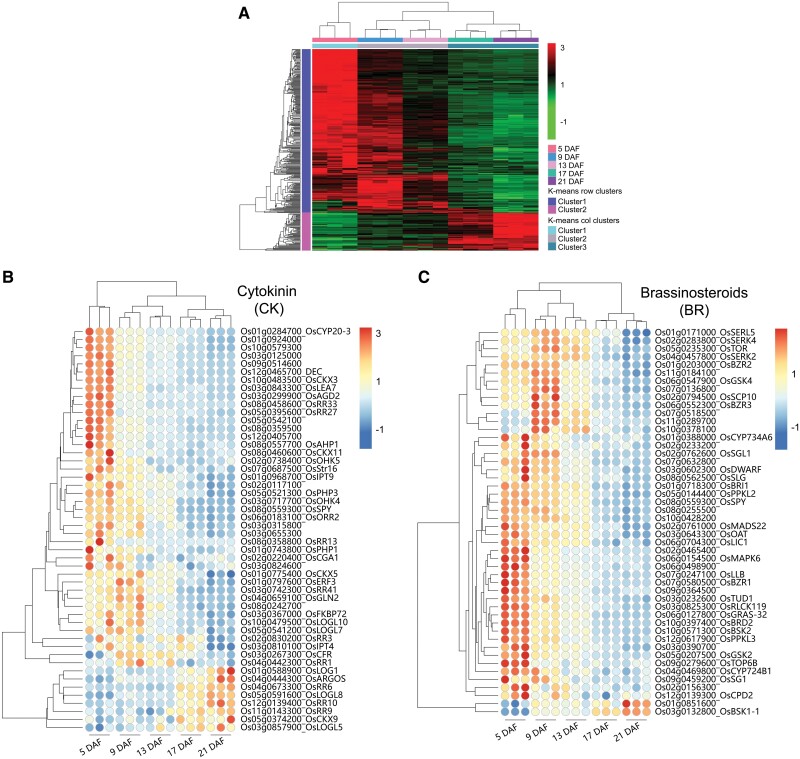

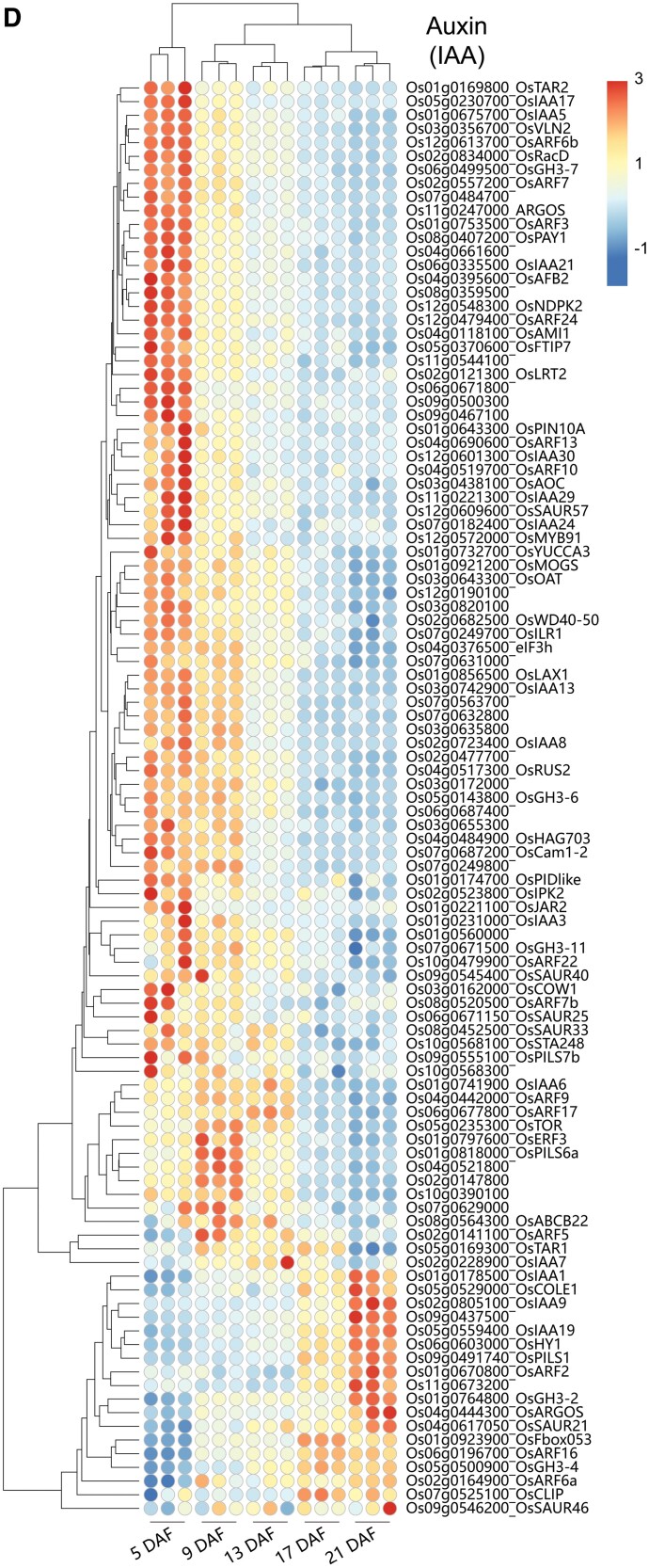

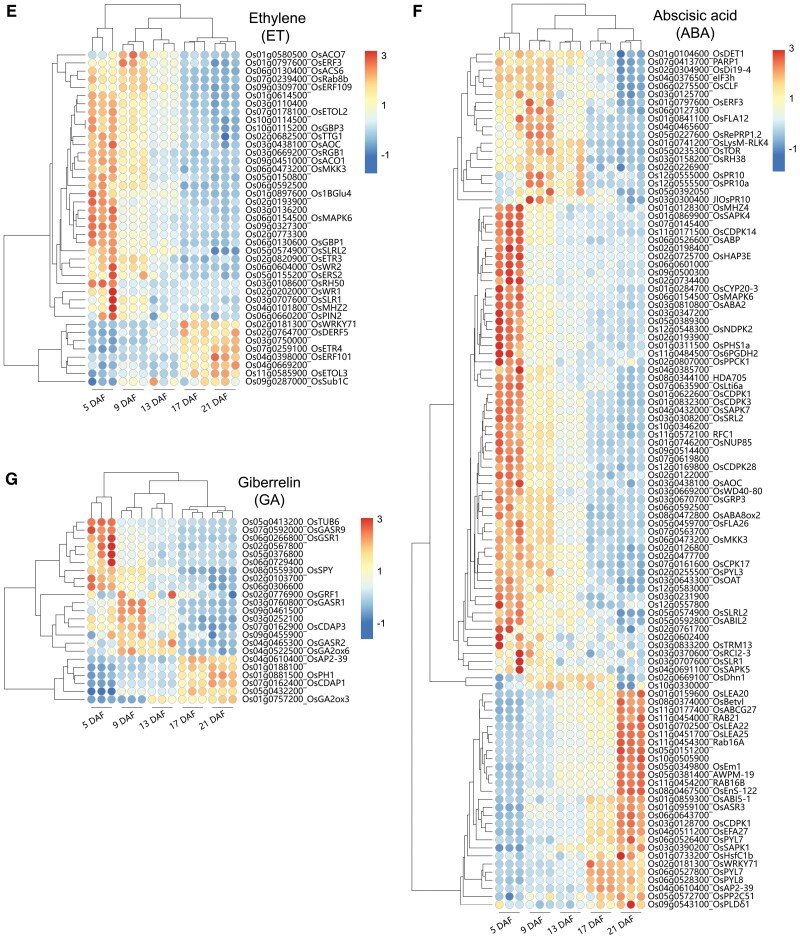
Expression profiles of genes related to hormone metabolism and transport in developing rice grain. (A) Hierarchical clustering of genes related to hormone metabolism and transport in profiles 0, 19, and 16 based on *Z*-score FPKM data. Clustering is calculated using the Euclidean correlation and average linkage. (B) Genes related to cytokinin (CK) metabolism, transporters, and receptors. (C) Genes related to brassinosteroid (BR) metabolism, transporters, and signaling. (D) Genes related to auxin (IAA) biosynthesis, signaling, and transporters. (E) Genes related to ethylene (ET) metabolism, transporters, and receptors. (F) Genes related to abscisic acid (ABA) signaling. (G) Genes related to gibberellin (GA) biosynthesis, signaling, and transporters. The gene-normalized signal intensities are shown in the heat maps using *Z*-scores. DAF, days after fertilization.

Because plant hormones play important roles in regulating grain development (Jones and Setter, 2000), we analyzed the expression pattern of genes in profiles 0, 19, and 16, which related to the biosynthesis and metabolism of auxin (indole-3-acetic acid, IAA), gibberellin (GA), brassinosteroid (BR), cytokinin (CK), abscisic acid (ABA), and ethylene (ET) (Fig. 4). Cluster analysis based on temporal changes in gene expression showed two clusters, one with high expression at 5–13 DAF and the other at 17–21 DAF (Fig. 4A). Among the first cluster, a large number of genes began to decline after being strongly expressed at 5 DAF. A few genes highly expressed at 9 and 13 DAF form the second cluster. The genes with high expression at 17 and 21 DAF form the third cluster. (Fig. 4A). In general, CK-, IAA-, ET-, BR-, ABA-, and GA-related genes showed three significant expression patterns in developing grains, with the majority of the genes being substantially expressed at 5–13 DAF, particularly at 5 DAF. For instance, many CK biosynthesis-related genes including *OsRR3*, *OsRR13*, *OsRR27*, and *OsRR33* were preferentially expressed at 5 DAF and showed reduced expression at 17 and 21 DAF (Fig. 4B). Only a few BR biosynthesis-related genes were expressed at 17–21 DAF (Fig. 4C). The genes encoding IAA biosynthesis enzymes, including *OsYUCCA3*, were mainly expressed at 5–13 DAF. Genes encoding IAA transporters, such as *Aux*/*IAA*, were expressed at higher levels at 5 DAF than at the later stages (Fig. 4D). Genes encoding ET signal transduction genes were mainly expressed at 5–13 DAF (Fig. 4E). A large number of genes involved in ABA biosynthesis and signaling were enriched in early developmental stages (Fig. 4F). *GA20ox* encodes a key enzyme in GA biosynthesis and, among GA20ox-encoding genes in rice, *GA20ox3* and *GA20ox6* were respectively expressed at 13–21 and 9–13 DAF (Fig. 4G). Only a few genes were strongly expressed at 17–21 DAF (Fig. 4).

**Fig. 5.**
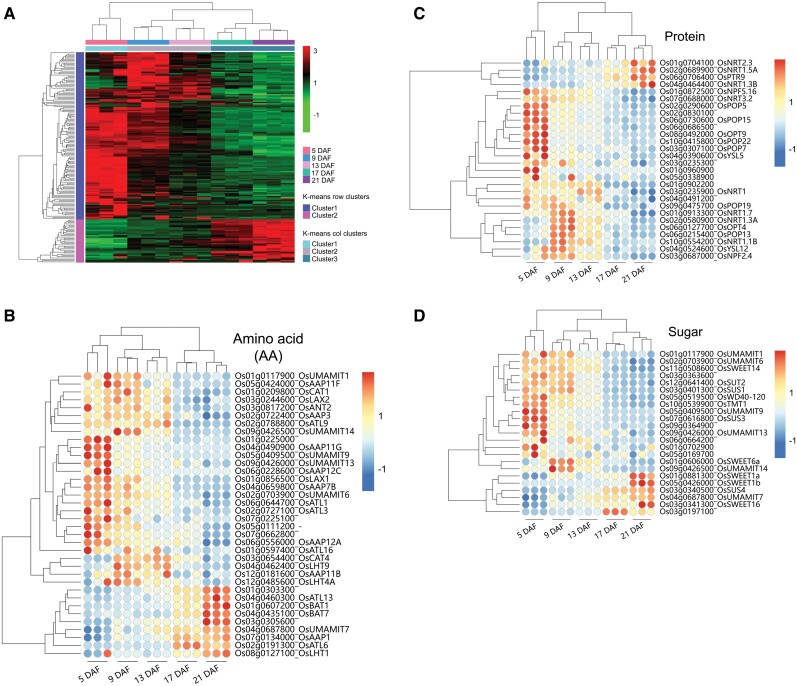

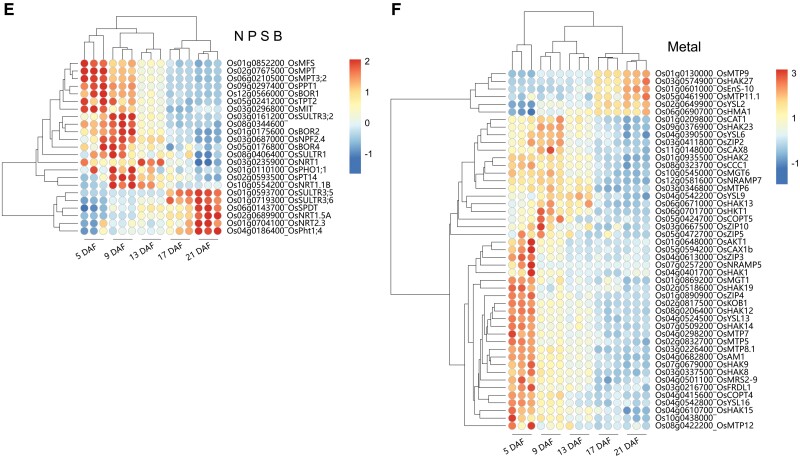
Expression profiles of genes related to nutrient syntheses and transporters. (A) Hierarchical clustering of genes related to transporters in profiles 0, 19, and 16 based on *Z*-score FPKM data. Clustering is calculated using the Euclidean correlation and average linkage. (B) Genes involved in amino acid transport. (C) Genes involved in protein transport. (D) Genes involved in sugar transport. (E) Genes involved in nitrate (N), phosphate (P), sulfate (S), and boron (B) transport. (F) Genes involved in metal ion transport. The gene-normalized signal intensities are shown in the heat maps using *Z*-scores. DAF, days after fertilization.

Anatomically, rice filial tissues including the endosperm and the embryo are symplastically isolated from the tissues of the mother plant and thus nutrient loading onto developing rice grains requires both efflux transporters on the maternal tissue side and influx transporters on the filial side (Krishnan and Dayanandan, 2003; Palmgren *et al.*, 2008). Genes encoding transporters for amino acids, proteins, sugars, nitrate, phosphate, sulfate, and metal ions are therefore likely to be important for grain filling. We performed cluster analysis on the transporter genes in profiles 0, 19, and 16. Overall, the majority of genes encoding nutrient transport were mainly expressed at 5–13 DAF and generally had a low expression activity at 17–21 DAF (Fig. 5). Most of the amino acid permease (AAP) genes, including *OsAAP3*, *OsAAP7B*, *OsAAP11B OsAAP11F*, *OsAAP11G*, and *OsAAP12C*, were preferentially expressed at high levels at 5–13 DAF, but *OsAAP1*, *OsATL6*, and *OsLHT1* showed a stronger expression at 21 DAF (Fig. 5B). The known genes encoding sucrose transporters, including *OsSUS1*, *OsSUS3*, *OsSUT2*, *OsTMT1*, and *OsSWEET14* were substantially expressed at 5–13 DAF, especially at 5 DAF (Fig. 5D). Interestingly, the expression patterns of transporters of specific nutrients are similar to those of their accumulation in the grain. Seven *KT/KUP/HAK* family genes (*OsHAK1*, *OsHAK8*, *OsHAK9*, *OsHAK12*, *OsHAK14*, *OsHAK15*, and *OsHAK19*) and *OsCAX1b* were highly expressed at 5 DAF but slightly decreased afterwards. This pattern was consistent with the K and Ca accumulation during the filling stage. Three nitrate transporter genes (*OsNPF2.4*, *OsNRT1.1B*, and *OsNRT1*), two phosphate transporter genes (*OsPT14* and *OsPHO1;1*), two sulfate transporter genes (*OsSultr1* and *OsSultr3;2*), two Mg^2+^ transporters (*OsMGT6* and *OsMRS2-6*), two ZRT- and IRT-like protein genes (*OsZIP2* and *OsZIP5*), and a Cu^2+^ transporter gene (*OsCOPT5*) were highly expressed during the middle of the filling stage (9–13 DAF) when the accumulation of these elements (including N, P, S, Mg, Mn, Cu, and Zn) reached the maximum rate (Fig. 5C, E, F). In contrast, a few transporter genes of these elements were also expressed at 17–21 DAF, such as *OsYSL2* encoding a metal–nicotianamine transporter, which was mainly expressed at 21 DAF, presumably contributing to the accumulation of Fe in the later filling stage (Figs 2, 5F).

### Redistribution of mineral elements from vegetative tissue during grain filling

**Fig. 6.**
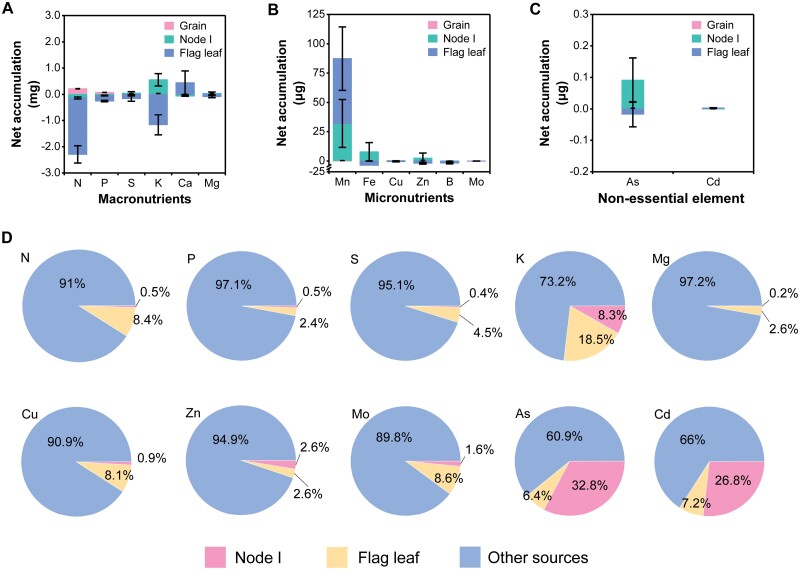
Redistribution of elements from vegetative tissues to grain. (A–C) Net accumulation content of macronutrients (A), micronutrients (B), and non-essential (toxic) elements (C) in the flag leaf, node I, and grain during grain filling. (D) Relative contribution of the flag leaf, node I, and other sources to the grain. Node I is the first node from top to bottom in the stem of rice plant. Other sources include plant tissues other than the flag leaf and node I, such as stem transport and root absorption. Data at different filling stage of vegetative tissue were obtained from three biological replicates; each biological replicate is from an individual plant. Data are means ±SD (*n*=3).

We assessed the redistribution of elements that had accumulated in the vegetative tissues prior to grain maturation. For this, the net accumulation index was used to estimate the contribution of redistribution, with a negative index indicating that elements were transported outward from the vegetative tissues during grain filling. Net negative accumulation indices were found for N, P, S, K, Mg, Fe, Cu, Zn, B, Mo, and As in the flag leaves, suggesting that these elements were redistributed from the flag leaves to the grain (Fig. 6A–C).

Based on principles of plant nutrition of mineral nutrient mobility in phloem (White, 2012), we assessed the contribution rates of 10 elements (N, P, S, K, Mg, Cu, Zn, Mo, As, and Cd) in vegetative tissues. Among the 10 elements, the largest contributions from the flag leaf to the total grain concentration were for K (18.5%) (Fig. 6D). Apart from vegetative tissues, node I, a transportation hub, plays a role in controlling the redistribution of elements (Yamaguchi *et al.*, 2012), and thus the contributions from node I to the total grain concentration were small for all elements (0.2–8.3%) except As and Cd (Fig. 6D). Generally, other sources (such as stem transport and root absorption) accounted for the majority of the accumulation of elements in grains (Fig. 6D).

### The spatial variation of elements in different branches within a panicle

**Fig. 7.**
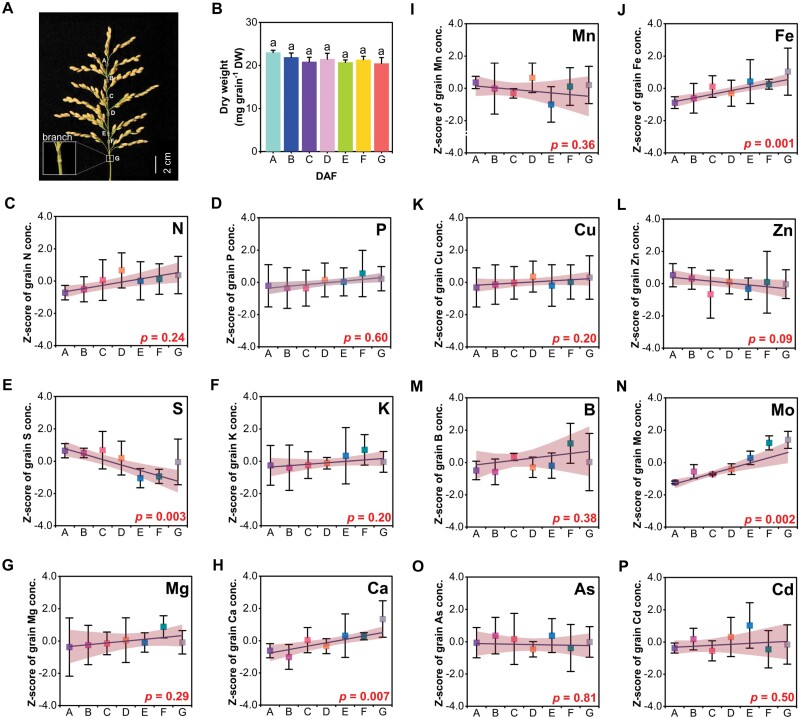
Grain concentration of different elements (*y*) in different parts of a panicle (*x*). (A) Schematic diagram of space division of a panicle. (B) Dry weight from the top to the bottom of the panicle. (C–P) Mineral element from the top to the bottom of the panicle. Data at different parts of a panicle were obtained from three biological replicates; each biological replicate consisted of 5–10 grains collected from three independent panicles. The *Z*-score method based on the mean and SD of the original data was used to standardize the grain element concentration, and the mean value of the processed data was 0 and the variance was 1. Lines represent linear regressions (equations and *R*^2^ are shown in Supplementary Table S5); Pearson correlation coefficient was used to measure the degree of correlation. Palevioletred is the 95% confidence interval, which was calculated using *t*-distributions based on sample variance and population mean. Scale bar=2 cm.

We divided the panicle from the top to the bottom into seven racemes (A–G) (Fig. 7A). There was no significant difference in the dry weight per grain among different racemes (Fig. 7B). In contrast, elemental concentrations varied among different racemes in three patterns. The concentrations of Ca, Fe, and Mo increased significantly from the top to the bottom, with grain concentrations in the bottom raceme being 51% higher than those in the top raceme for Ca, 94% higher for Fe, and 46% higher for Mo (Fig. 7G, I, M). In contrast, the S concentration in the grain at the top was 8% higher than that in the grain at the bottom (Fig. 7D). In the third pattern, no significant differences were observed for the concentrations of P, K, Mg, Mn, Cu, Zn, B, As, and Cd.

**Fig. 8.**
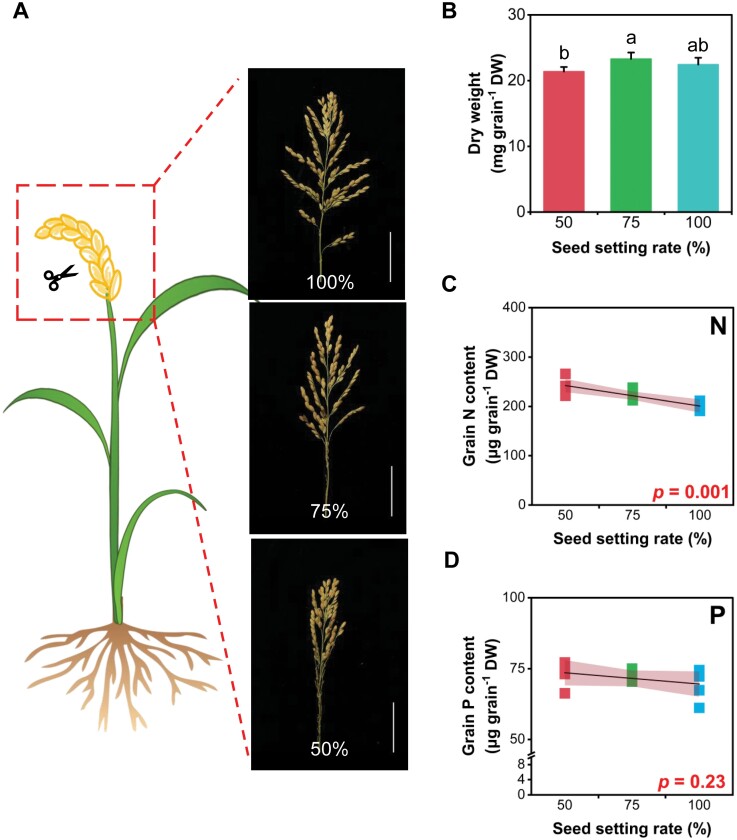

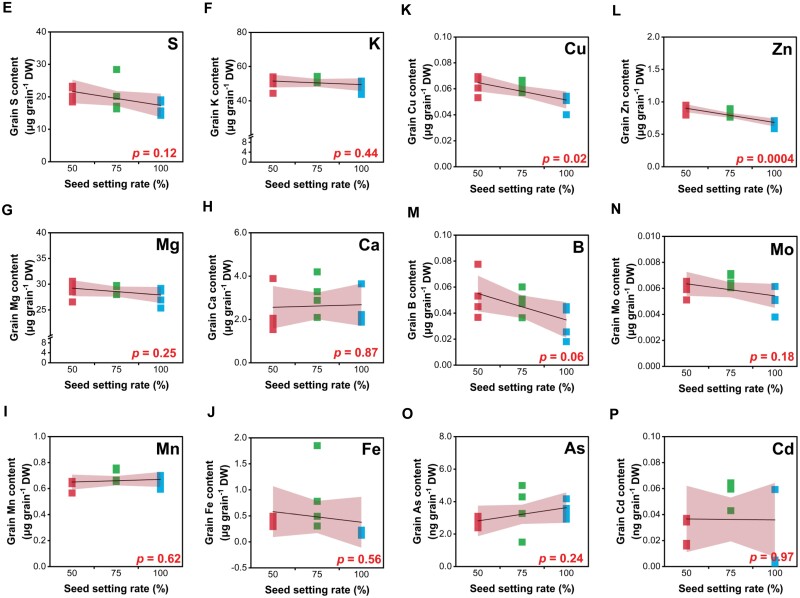
Elemental accumulation in grains in response to altered sink–source relations. (A) Schematic diagram of different seed setting rates. (B) Grain dry weight at different seed setting rates. (C–P) Contents of elements in grains at different seed setting rates. At the heading stage, the base of the main panicle was pruned at heading stage to control the rice seed setting rate at 50, 75, and 100% artificially. Data were obtained from four biological replicates; each biological replicate consisted of 10 grains collected from four independent panicles. Lines represent linear regressions (equations and *R*^2^ are shown in Supplementary Table S6); Pearson correlation coefficient was used to measure the degree of correlation. Palevioletred is the 95% confidence interval, which was calculated using *t*-distributions based on sample variance and population mean. Scale bars=4 cm.

We further assessed the effect of seed setting rate on grain element accumulation. It was found that the grain contents of N, Cu, and Zn correlated significantly and negatively with seed setting rates (N, Zn, and Cu concentrations in grains decreased by 175–38% with the increase of seed setting rate) (Fig. 8J, K). For the remaining elements, no significant changes were observed across the seed setting rates.

### Element accumulation pattern mediates spatial distribution differences

**Fig. 9.**
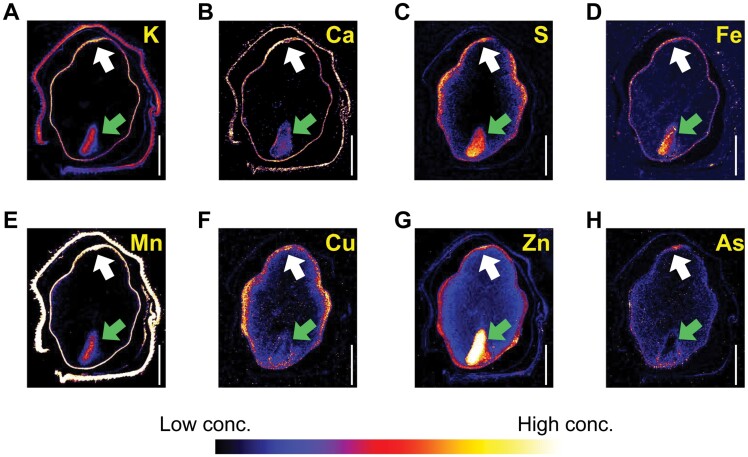
Distribution of elements [K (A), Ca (B), S (C), Fe (D), Mn (E), Cu (F), Zn (G), and As (H)] in a transverse section of a rice grain analyzed using μ-XRF. The section was 100 μm thick. Scale bar=1.0 mm. White arrows point to the dorsal stylar vascular trace; green arrows point to the embryo. As distribution (H) is from Tang *et al.* (2020). Dimethylarsinic acid is the causal agent inducing rice straighthead disease. Journal of Experimental Botany 71, 5631–5644, with permission from the Society for Experimental Biology.

We used synchrotron-based µ-XRF to determine the distribution patterns of various elements in transverse sections of grain (Fig. 9). The concentrations of K, Ca, and Mn, belonging to the ‘early accumulation pattern’ (Fig. 2A, C, D), were strongly accumulated in the husk and highly localized in the pericarp/aleurone layer (Fig. 9A, B, E). In contrast, the concentrations of S, Fe, Cu, Zn, and As were highest in the aleurone/pericarp layer and decreased gradually from the outer parts of the starchy endosperm toward the interior of the endosperm. S and Cu had a similar pattern except for the embryo (Fig. 9C, F). S, Fe, and Zn were co-located in the aleurone layer and embryo (Fig. 9C, D, G). Almost all elements had higher concentrations in the dorsal stylar vascular trace (white arrow in Fig. 9), whilst K, S, Fe, Mn, and Zn were highly localized in the embryo (green arrow in Fig. 9).

## Discussion

### The gene expression patterns were similar to the nutrient accumulation models at the filling stage

Three patterns for both elemental accumulation and gene expression in grains were identified: (i) early accumulation/response pattern; (ii) middle accumulation/response pattern; and (iii) sustained increase accumulation/response pattern (Figs 2 and 3). The analyses of the trend cluster revealed three important periods during the grain-filling process [0–5 DAF, 9–13 DAF, and 17–21 DAF (or maturation)] (Fig. 1A) (Krishnan and Dayanandan, 2003; Itoh *et al.*, 2005; Wu *et al.*, 2016b; An *et al.*, 2020). The first stage is dominated by the morphogenesis of the embryo (Itoh *et al.*, 2005). A large number of genes involved in transcriptional activity, DNA replication, protein biosynthesis, and hormone metabolism, and transport genes were expressed during early embryo development (Figs 3A–C, 4; Supplementary Fig. S5). For instance, multiple RPs had significantly higher transcript abundance at 5 DAF (Supplementary Fig. S5). The *Aux/IAA* gene is important in signaling, involved in many developmental processes, such as vascular tissue formation, adventitious root initiation, tropistic responses, apical dominance, and flower and fruit development andcoordinating the organization of the embryo by providing positional information (Fig. 4D) (Chen *et al.*, 2014). K was preferentially accumulated during the early stage of developing grains, making a substantial contribution to maintaining the cellular electroneutrality and osmotic equilibrium (Kochian and Lucas, 1989; Rodríguez-Navarro, 2000). Seven *KT/KUP/HAK* family genes (*OsHAK1*, *OsHAK8*, *OsHAK9*, *OsHAK12*, *OsHAK14*, *OsHAK15*, and *OsHAK19*) were strongly expressed at 5 DAF, suggesting that they may be involved in K accumulation at the initial stage of grain filling (Figs 2A, 5F). Ca and B also accumulated early, probably to stabilize the cytoderm structure (Oetting *et al.*, 1993; Sanders and Brownlee, 2002). We identified that the expression level of *OsCAX1b* and three efflux boron transporter genes (*OsBOR1*, *OsBOR2*, and *OsBOR4*) coincided with the rapid Ca and B accumulation at 5 DAF (Figs 2B, D, 5D). The rice caryopsis was green during the early stage (Fig. 1A) and each cross cell contains 2–5 chloroplasts (Krishnan and Dayanandan, 2003; Chernev *et al.*, 2021). The high expression of Mn transporter genes (e.g. *OsYSL6*) at 5 and 9 DAF probably reflects the requirement of Mn for PSII in the chloroplast (Fig. 5F).

Most morphogenetic events are completed before the second stage (9–13 DAF). The second stage had a typical feature of elevated nutrient accumulation (Fig. 2), during which the embryo and endosperm become symplastically isolated so that nutrients are moved from the endosperm to the developing embryo via apoplastic transportation for supporting embryo development (Jones and Rost, 1989; Itoh *et al.*, 2005; An *et al.*, 2020). For example, sucrose is the main form of carbohydrates transported from the maternal tissues to the endosperm and is secreted from the endosperm to feed the embryo by SUTs (for sucrose) and SWEETs (for hexose and sucrose). *OsSUT2* and *OsSWEET14* were highly expressed in the starch-filling milky phase, which coincided with the onset of rapid biomass accumulation (Fig. 5D). In addition, we found different expression patterns of *SWEET* genes during different stages of grain development. For example, *OsSWEET14* was mainly expressed during the rapid grain-filling stage (5–13 DAF), and Fei *et al.* (2021) confirmed that *OsSWEET14* was strongly expressed in cross cells. Conversely, *OsSWEET1a*, *OsSWEET1b*, and *OsSWEET16* were expressed more highly at 21 DAF, suggesting that different apoplasmic pathways supply sucrose to the endosperm during the rapid grain-filling stage in rice via SWEET sucrose effluxers (Fig. 5D) (Fei *et al.*, 2021). In most plants, organic N is primarily transported in the form of amino acids (Lam *et al.*, 1996). Some members of the 'usually multiple acids move in and out transporter’ (UMAMIT) family, and amino acid transporter (AAPs) might mediate amino acid export from endosperm to the developing embryo (Sanders *et al.*, 2009; Besnard *et al.*, 2018). However, further investigations including tissue expression and subcellular localization of the gene products are required to confirm the role of these transporters.

From 9 to 13 DAF is the key period for the continuous unloading of minerals from the maternal tissues to form viable seeds, as evidenced by the onset of rapid element accumulation (Fig. 2). Many divalent cation transporters (i.e. Mg^2+^, Zn^2+^, and Cu^2+^) had higher transcriptional levels at 9–13 DAF in developing grain, which coincided with the pattern of their element accumulation (Figs 2H–J, 5D). Additionally, *OsYSL2* and *OsYSL9* were Fe-chelate transporter genes. OsYSL2 transports Fe(II)–NA, while OsYSL9 transports Fe(II)–NA and Fe(III)–DMA, which were mainly expressed at 13–21 DAF (Fig. 5D; Supplementary Fig. S6). Strong *GUS* (β-glucuronidase) staining of *OsYSL2* was increased in the vascular bundles, embryo, and peripheral layer of the endosperm after fertilization until maturity, while *OsYSL9* was expressed at the interface between embryo and endosperm, suggesting that they probably participate in the intercompartmental Fe homeostasis or distribution within rice seed (Fig. 9D) (Koike *et al.*, 2004; Senoura *et al.*, 2017). Both Fe and Zn preferentially accumulate in the outer layer of grain (Fig. 9), probably due to their association with phytates. It has been shown that the phosphate transporter gene *OsPHO1;1* was expressed in the ovular vascular trace and the outer layer of the inner integument, while *OsPHO1;2* was expressed in the nucellar epidermis of caryopses (Chorpet *et al.*, 2016; Ma *et al.*, 2021). Taken together, these results suggest that expression of some transporter genes was associated with the onset of rapid nutrient accumulation, such as many K^+^ transporter genes more highly expressed at 5 DAF. Some genes expressed in specific tissues such as *OsYSL2* and *OsYSL9* may mediate the final distribution of Fe within grain tissues.

### Source of elements during grain filling

There are two sources of mineral supply during grain development: (i) direct uptake from the soil, and (ii) redistribution of stored minerals in vegetative tissues as they senesce during grain filling. The elements with a high mobility, such as K, are more easily remobilized from vegetative tissues to the grain (Fig. 6A, D). In contrast, Ca and Mn have low phloem mobility and thus the demand from growth sinks must be met by the xylem rather than by redistribution (Nable and Loneragan, 1984; Jeschke and Pate, 1991). Our results confirm that the flag leaf was acting as a sink (rather than a source) for these two elements because concentrations increased in the flag leaf during the filling stage (Fig. 6A, B), suggesting that both root absorption and xylem translocation were important for their accumulation in the grain. Our results also show that root absorption was still the main pathway of grain mineral nutrient accumulation during the filling stage, although the availability of elements, except Cd, in the soil was reduced due to soil drainage during the later stage of filling (Supplementary Fig. S7). As and Cd, two non-essential elements, are redox-sensitive elements. The solubility of As increases dramatically as redox potential decreases once a paddy soil is flooded, primarily due to the reductive dissolution of iron (oxy)hydroxides and reduction of As(V) to As(III), with the latter less strongly sorbed than As(V). In contrast, solubility of Cd in paddy soils decreases as redox potential decreases upon flooding as a result of the formation of insoluble CdS and increased pH in the acidic soils which promotes Cd sorption. When paddy water is drained, the reverse processes occur rapidly, resulting in increased Cd solubility but decreased As solubility. Therefore, they have a high risk of transfer from paddy soil to rice grain due to the mobility altered upon flooding or draining conditions (Arao *et al.*, 2009; Meharg *et al.*, 2009, 2013; Li *et al.*, 2011; Zhao and Wang, 2020). Soil redox potential (Eh) was found to be an important factor influencing Cd availability in soils (Fulda *et al.*, 2013; Furuya *et al.*, 2016; Wang *et al.*, 2019; H. Huang *et al.*, 2021). Our soil incubation showed that Cd availability increased markedly once soil redox potential increased after paddy soil drainage, which was synchronous with its accumulation pattern in the grain (Fig. 2M; Supplementary Fig. S7). Conversely, soil As solubility decreased substantially during the grain-filling period (Supplementary Fig. S7), but this change in As availability was not synchronous with its accumulation pattern in the grain (Fig. 2L). The concentration of Cd in grains did not increase over time in our data, with this attributable to the dilution from the increase in dry matter accumulation, very low soil Cd concentration (0.06 mg kg^–1^), and near-neutral pH (7.15) (Fig. 2M; Supplementary Fig. S7; Supplementary Table S1). Our results showed that Cd uptake from the root to the grain is an important source of grain Cd post-flowering except Cd redistribution in vegetative tissues (Fig. 6D). Furthermore, the nodes are likely to be key locations of xylem to phloem transfer in the pathway from the root to the grain during the grain-filling stage because nodes contribute 26.8% to the final grain Cd content (Fig. 6D), with this consistent with previous results (Fujimaki *et al.*, 2010; Rodda *et al.*, 2011). The redistribution of As from flag leaves contributed to its accumulation in the grain (Fig. 6D). This result was consistent with a recent report by B.Y. Huang *et al.* (2021) who found that ~95% of the final grain As content was redistributed from other plant tissues that had accumulated in the plant prior to grain filling, whereas the contribution of root uptake of As during grain filling was negligible after the soil was drained, whilst ~98% of the grain Cd content was derived from the root uptake during grain filling. These results suggest that using a segmented water management strategy so as to avoid peak accumulation of elements can produce rice grain with both Cd and As concentrations below the food limits (Fulda *et al.*, 2013; Furuya *et al.*, 2016; Wang *et al.*, 2019; B.Y. Huang *et al.*, 2021).

### Spikelet development and sink capacity affect the elemental accumulation

Spikelet development is not synchronized among primary branches or even within a primary branch, and, as a result, the spikelets on the upper racemes form earlier than those on the lower racemes (Ikeda *et al.*, 2004; Itoh *et al.*, 2005). This difference could be one of the reasons why Fe, Mo, Ca, and S had a spatial variation within the same panicle (Fig. 7). In addition, the lower nutrient concentrations found in distal grains could be related to the transport of nutrients from the rachis to distal positions, nutrient mobility in the phloem (S>Fe>Mo>Ca), and the ratio of phloem versus xylem concentration of nutrients (Kochian, 1991; White, 2012). This variation has also been reported previously for other crops. For example, Huber *et al.* (2016) reported that seeds of soybean (*Glycine max*) produced at the top of the canopy had lower concentrations of minerals such as Fe compared with seeds produced at the bottom of the canopy. Calderini and Ortiz-Monasterio (2003) observed differences in concentrations of essential elements in wheat, with grain macronutrient and micronutrient concentrations decreasing from basal to more distal from the rachis, especially for Ca which decreased by 30%. The number of grains per panicle largely determines the sink capacity of the rice crop (Fukai *et al.*, 2008). Grain mineral nutrients including N, Cu, and Zn were more sensitive than single grain weight in response to the sink capacity reduction by spikelet removal; fewer seeds number per panicle had higher N, Cu, and Zn content in grains, indicating that the sink capacity has an important impact on element accumulation in rice (Fig. 8).

It should be noted that although transcriptome sequencing has provided a vast array of valuable data regarding expression profiles of the whole gene set across the grain developmental stages and our results found linkages between expression profiles and nutrient accumulation models at the filling stage, there is very little evidence for the role of these genes. Further studies are required to verify these linkages. In addition, the present study was carried out on soil with low concentrations of Cd and As and a neutral pH. The accumulation features of the two contaminants in rice grain may be different in contaminated and acidic soils, and further work is required in this regard. Finally, in the pruning test with different seed setting rates, we manually pruned spikelets at the bottom of the panicle. This bias may lead to the difference in grain elements at different seed setting rates.

In summary, the present study identified three distinct patterns for nutrient accumulation and three corresponding patterns of gene expression during the filling stage of rice grains. The different patterns of nutrient accumulation are likely to be associated with the biological functions of the elements, soil supply, redistribution, and gene expression patterns. Some key essential elements (i.e. N, P, S, Mg, Cu, Zn, and Mo) accumulated in a pattern similar to dry matter. Furthermore, the spatial distribution of elements on the same panicle was caused by the asynchrony of spikelet development and element mobility. These observations improve our knowledge of nutrient transport, redistribution mechanisms, and molecular processes during rice grain development. This information is useful for future functional genomics studies of rice grain development and grain filling as well as for the design of novel biofortification strategies.

## Supplementary data

The following supplementary data are available at *JXB* online.

Table S1. Background properties of the soil from the experimental site.

Table S2. Data filtering of reads.

Table S3. Primer sets used in the present study.

Table S4. Digestion programmer (for HNO_3_–HClO_4_).

Table S5. Linear regression equations of the *Z*-score of grain element concentration in different racemes.

Table S6. Linear regression equations of grain element content in different seed setting rates.

Fig. S1. Temporal changes in grain dry matter and the content of 14 elements within the grain.

Fig. S2. Concentrations of 14 elements in the rice grain over time.

Fig. S3. PCA plots with each point representing an independent biological replicate.

Fig. S4. Trend analysis of genes expressed in developing grains (5–21 DAF).

Fig. S5. Temporal dynamics of ribosomal protein family gene expression in developing grain (5–21 DAF).

Fig. S6. The expression levels of different ion transporter genes in developing grain (5–21 DAF)

Fig. S7. Temporal changes in soil soluble mineral elements in the soils.

erac426_suppl_Supplementary_Figures_S1-S7_Tables_S1-S6

## Data Availability

Raw sequence data of RNA-seq have been deposited in the NCBI SRA database, with accession numbers SRR17706479, SRR17709283, SRR17709378, SRR17709379, and SRR17709726.
