# Supplementary material for: Nutrient accumulation and transcriptome patterns during grain development in rice
Source: J Exp Bot. 2022 Oct 22;74(3):909–30. doi: 10.1093/jxb/erac426 (PMC9899419; doi:10.1093/jxb/erac426)
Supplement: erac426_suppl_Supplementary_Figures_S1-S7_Tables_S1-S6 [file erac426_suppl_supplementary_figures_s1-s7_tables_s1-s6.pdf]

Supplementary information

Nutrient accumulation and transcriptome patterns during grain development in rice (*Oryza sativa*)

Zi-Wen Ren[a], Peter M. Kopittke[b], Fang-Jie Zhao[a], Peng Wang[a,*]

Supporting Information:

7 Figures

6 Table

18 Pages

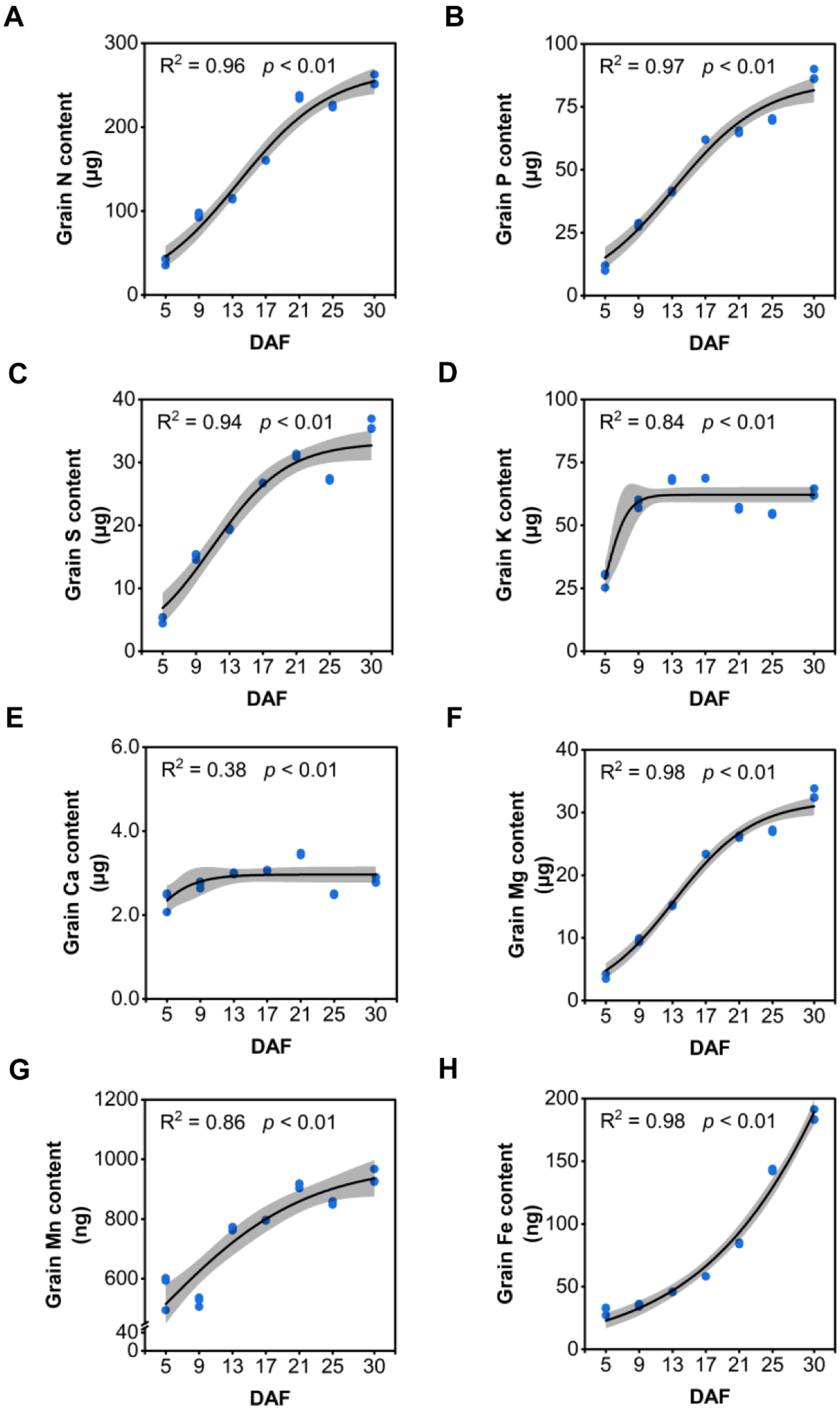
A
B
C
D
E
F
G
H
R² = 0.96 p < 0.01
R² = 0.97 p < 0.01
R² = 0.94 p < 0.01
R² = 0.84 p < 0.01
R² = 0.38 p < 0.01
R² = 0.98 p < 0.01
R² = 0.86 p < 0.01
R² = 0.98 p < 0.01
Grain N content (µg)
Grain P content (µg)
Grain S content (µg)
Grain K content (µg)
Grain Ca content (µg)
Grain Mg content (µg)
Grain Mn content (ng)
Grain Fe content (ng)
DAF

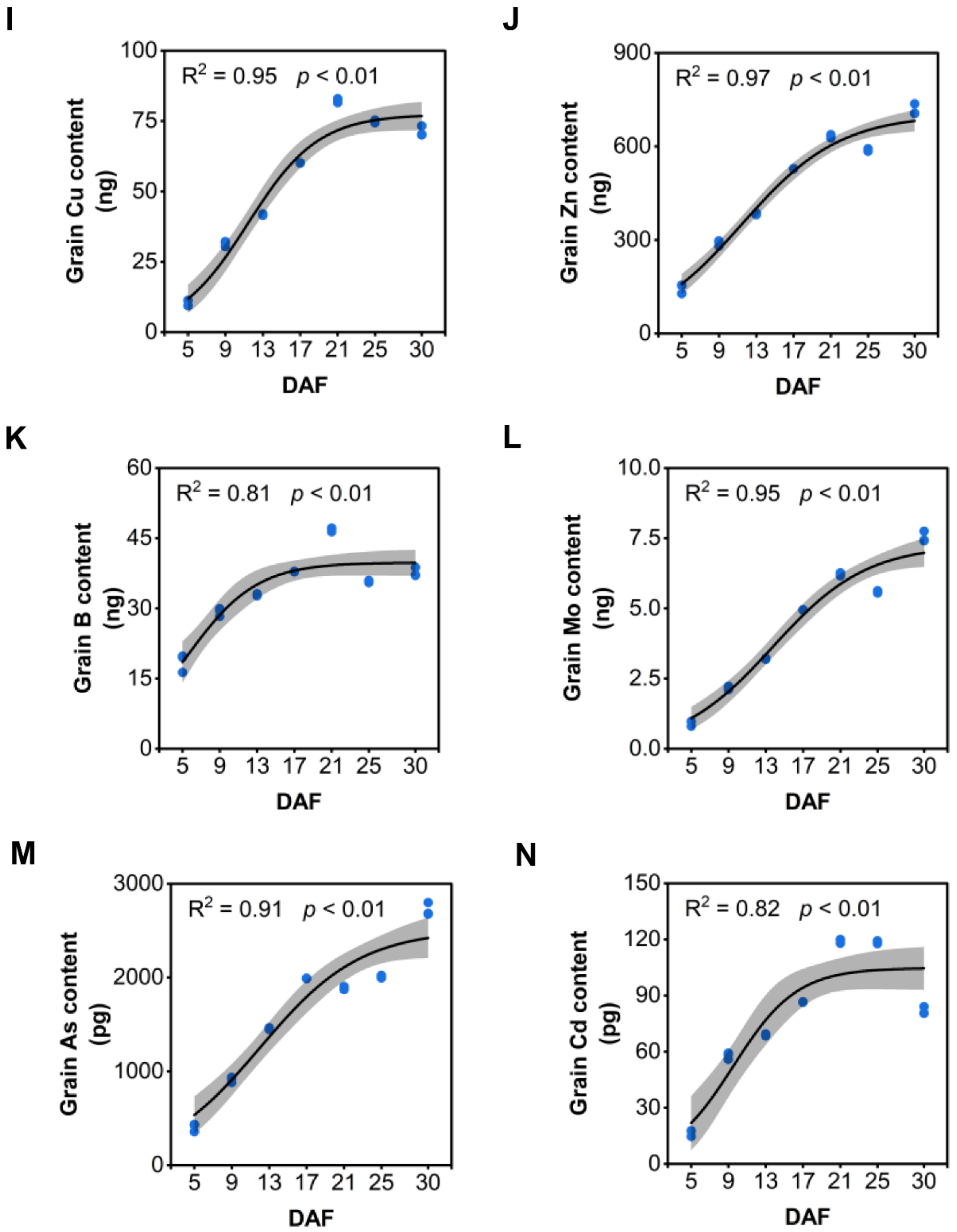

Figure S1. Changes in grain dry matter and the content of 14 elements within the grain over time. Data at different developmental stages were obtained from three biological replicates, each biological replicate consisted of 10-80 grains collected from marked panicles. Grey is the 95% confidence interval, which were calculated using *t*-distributions based on sample variance and population mean. DAF, days after fertilization.

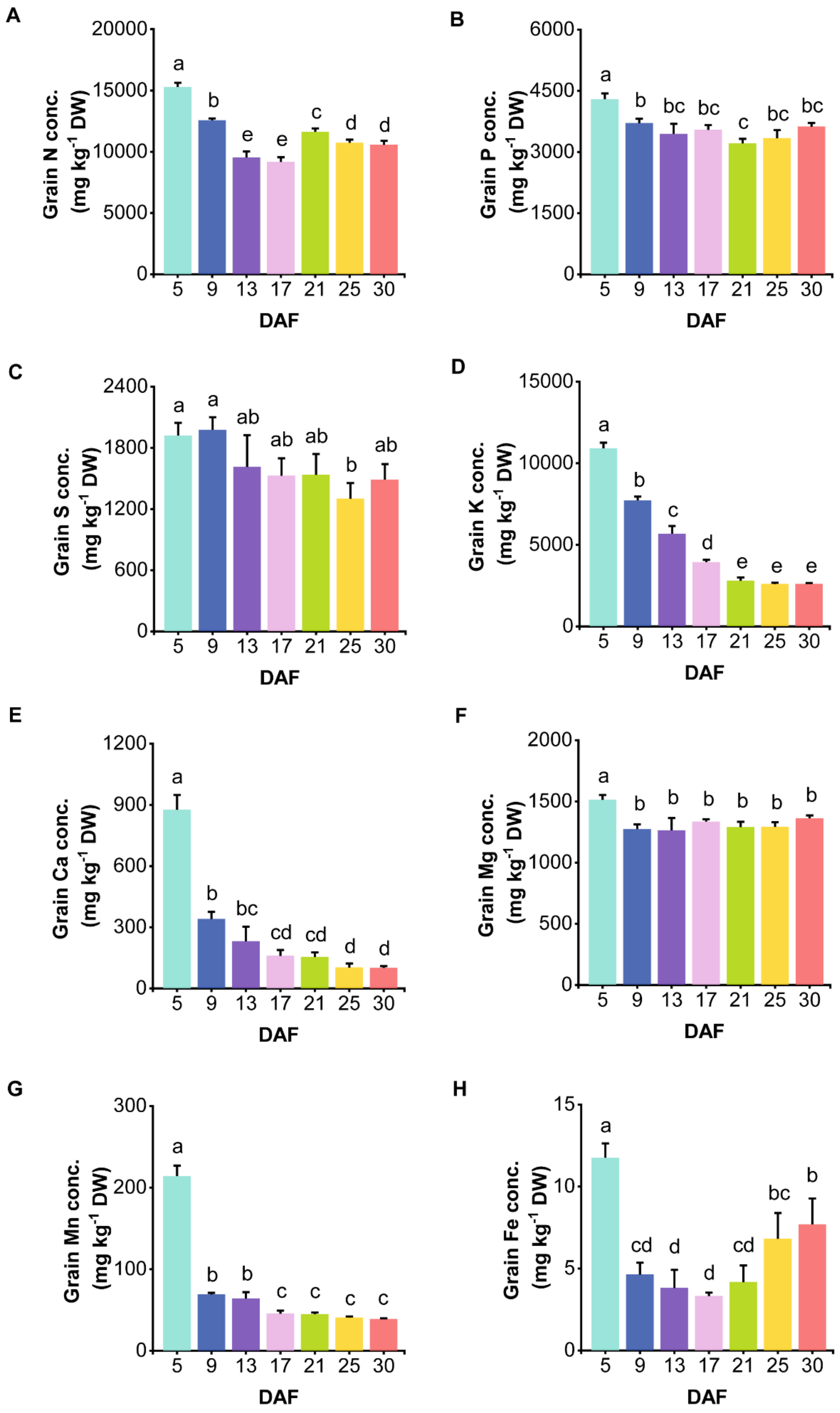
A
Grain N conc. (mg kg-1 DW)
20000
15000
10000
5000
0
a
b
e
e
c
d
d
5 9 13 17 21 25 30
DAF
B
Grain P conc. (mg kg-1 DW)
6000
4500
3000
1500
0
a
b
bc
bc
c
bc
bc
5 9 13 17 21 25 30
DAF
C
Grain S conc. (mg kg-1 DW)
2400
1800
1200
600
0
a
a
ab
ab
ab
b
ab
5 9 13 17 21 25 30
DAF
D
Grain K conc. (mg kg-1 DW)
15000
10000
5000
0
a
b
c
d
e
e
e
5 9 13 17 21 25 30
DAF
E
Grain Ca conc. (mg kg-1 DW)
1200
900
600
300
0
a
b
bc
cd
cd
d
d
5 9 13 17 21 25 30
DAF
F
Grain Mg conc. (mg kg-1 DW)
2000
1500
1000
500
0
a
b
b
b
b
b
b
5 9 13 17 21 25 30
DAF
G
Grain Mn conc. (mg kg-1 DW)
300
200
100
0
a
b
b
c
c
c
c
5 9 13 17 21 25 30
DAF
H
Grain Fe conc. (mg kg-1 DW)
15
10
5
0
a
cd
d
d
cd
bc
b
5 9 13 17 21 25 30
DAF

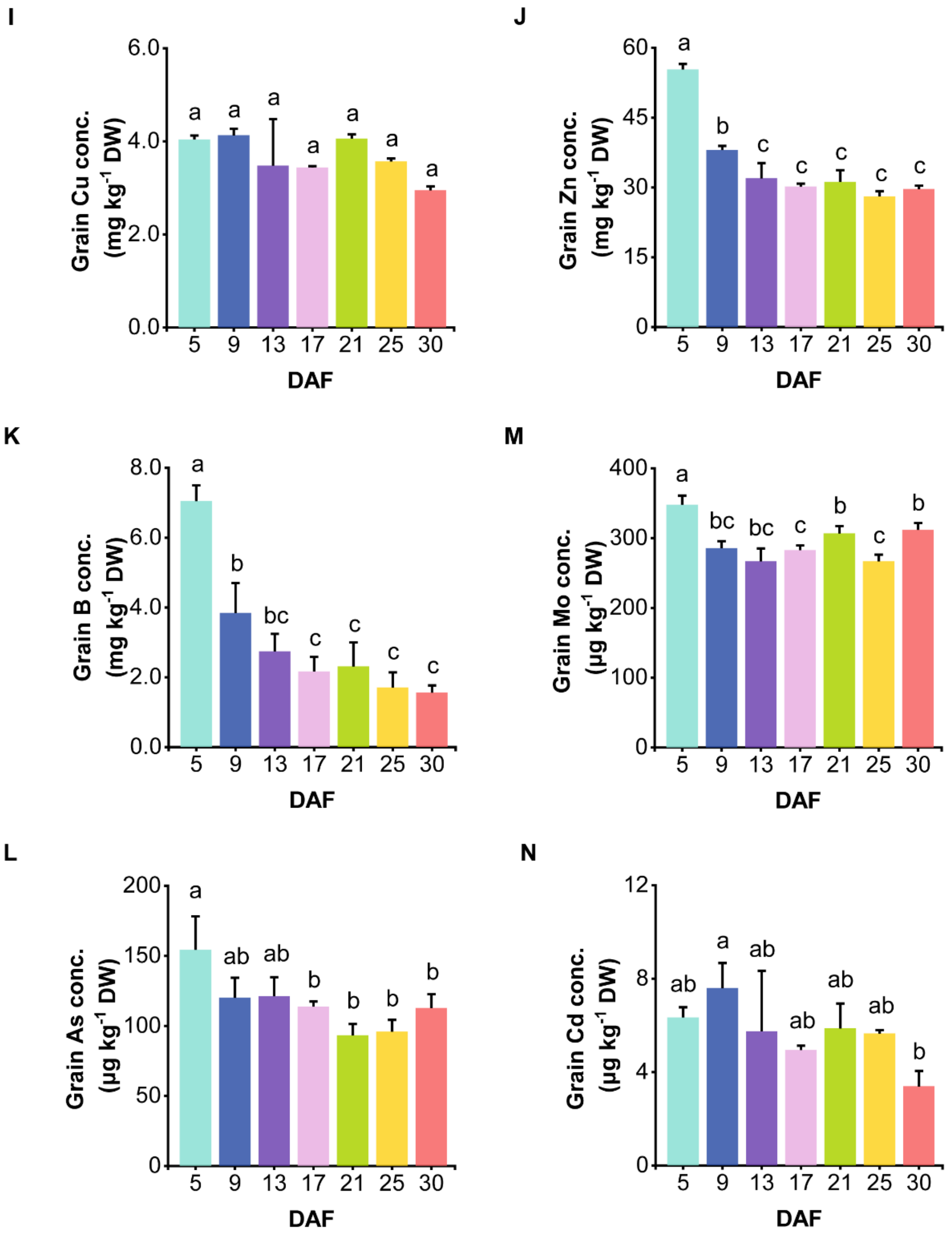

Figure S2. Concentrations of 14 elements concentration in the rice grain over time. Data at different developmental stages were obtained from three biological replicates, each biological replicate consisted of 10-80 grains collected from marked panicles. Different letters indicate significant differences among different developmental stages (Tukey's test, $P < 0.05$). DAF, days after fertilization.

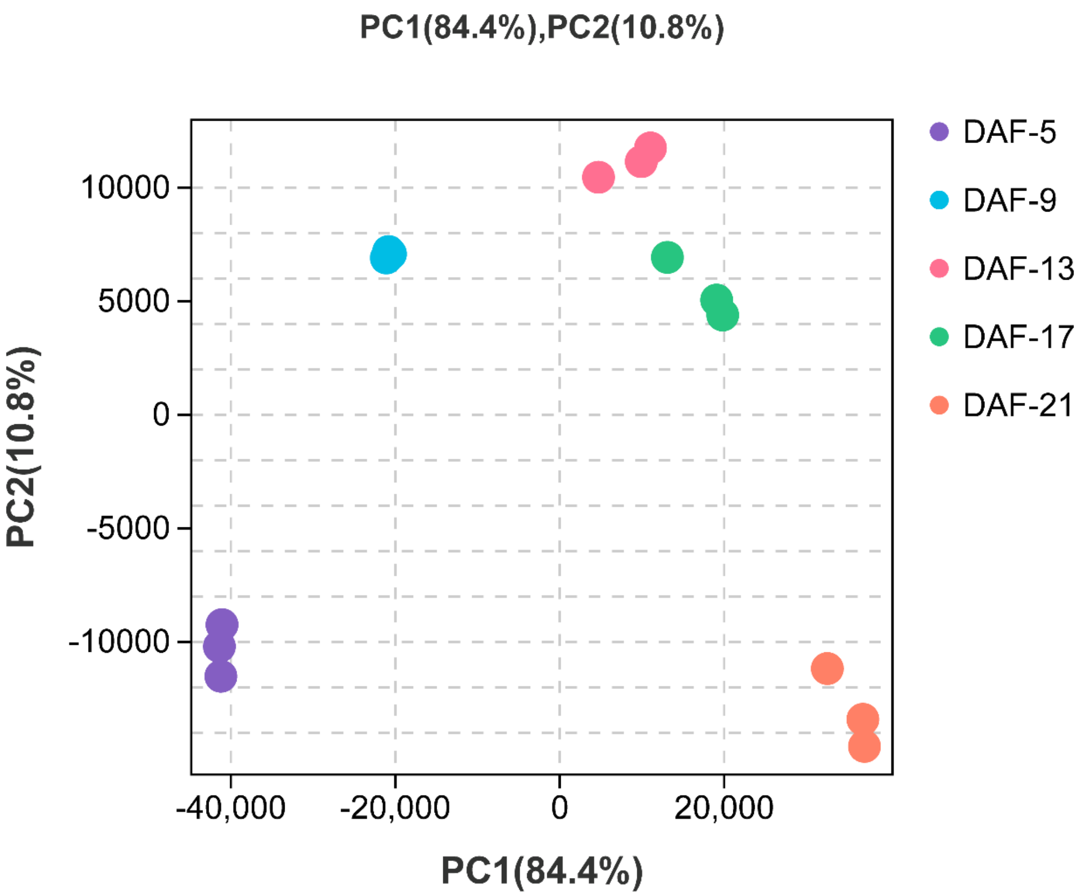

Figure S3. PCA plots with each point representing an independent biological replicate. DAF, days after fertilization.

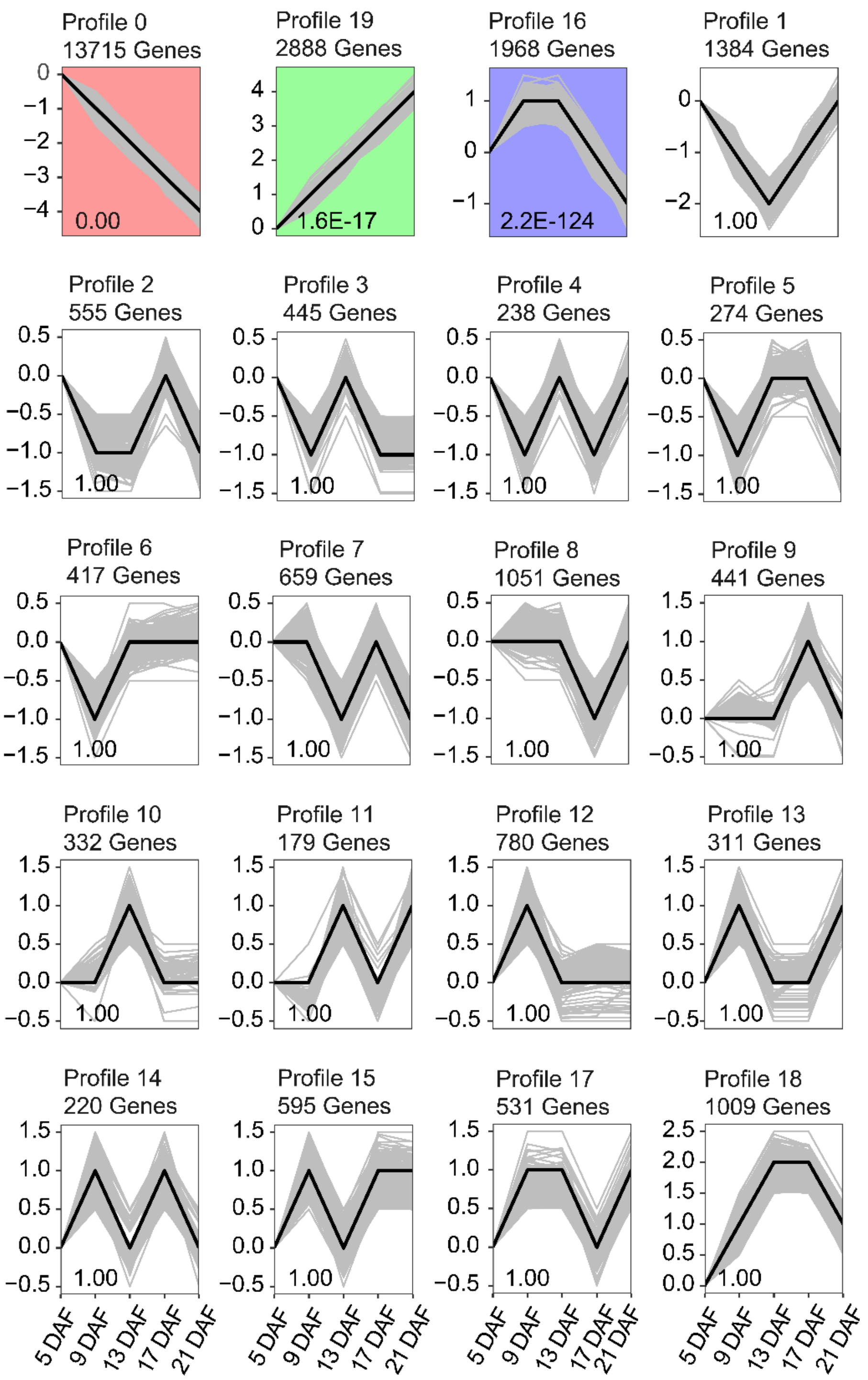

Figure S4. Trend analysis of genes expressed in developing grains (5-21 DAF). *P*-values are represented below each Profile. Each gray line represents a gene, and the bold black line represents dynamic expression patterns. Profiles ordered based on the *p*-value significance of number of genes assigned versus expected. Colors indicate three gene expression trends: red (#fe9999), early response pattern; green (#99fe99), sustained increase pattern; blue (#9999fe), middle response pattern.

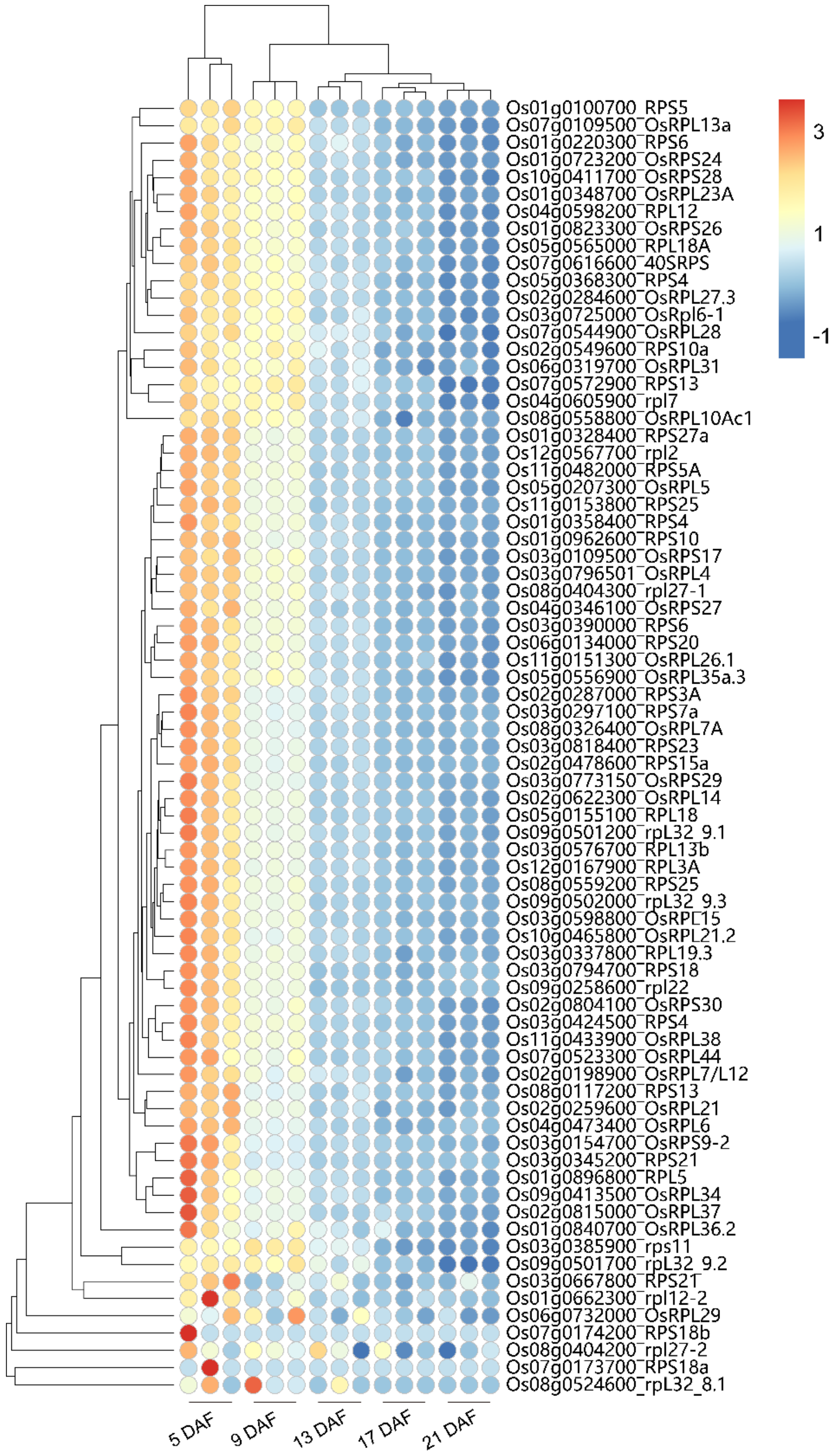

Figure S5. Temporal dynamics of ribosomal protein family gene expression in developing grain (5-21 DAF). The gene-normalized signal intensities are shown in the heat maps using Z-Scores, average linkage and euclidean methods were used for clustering. Change in color of the rectangle from red (#da382c) to blue (#3f71b2) represents a gradual decrease in the gene expression abundance. DAF, days after fertilization.

A

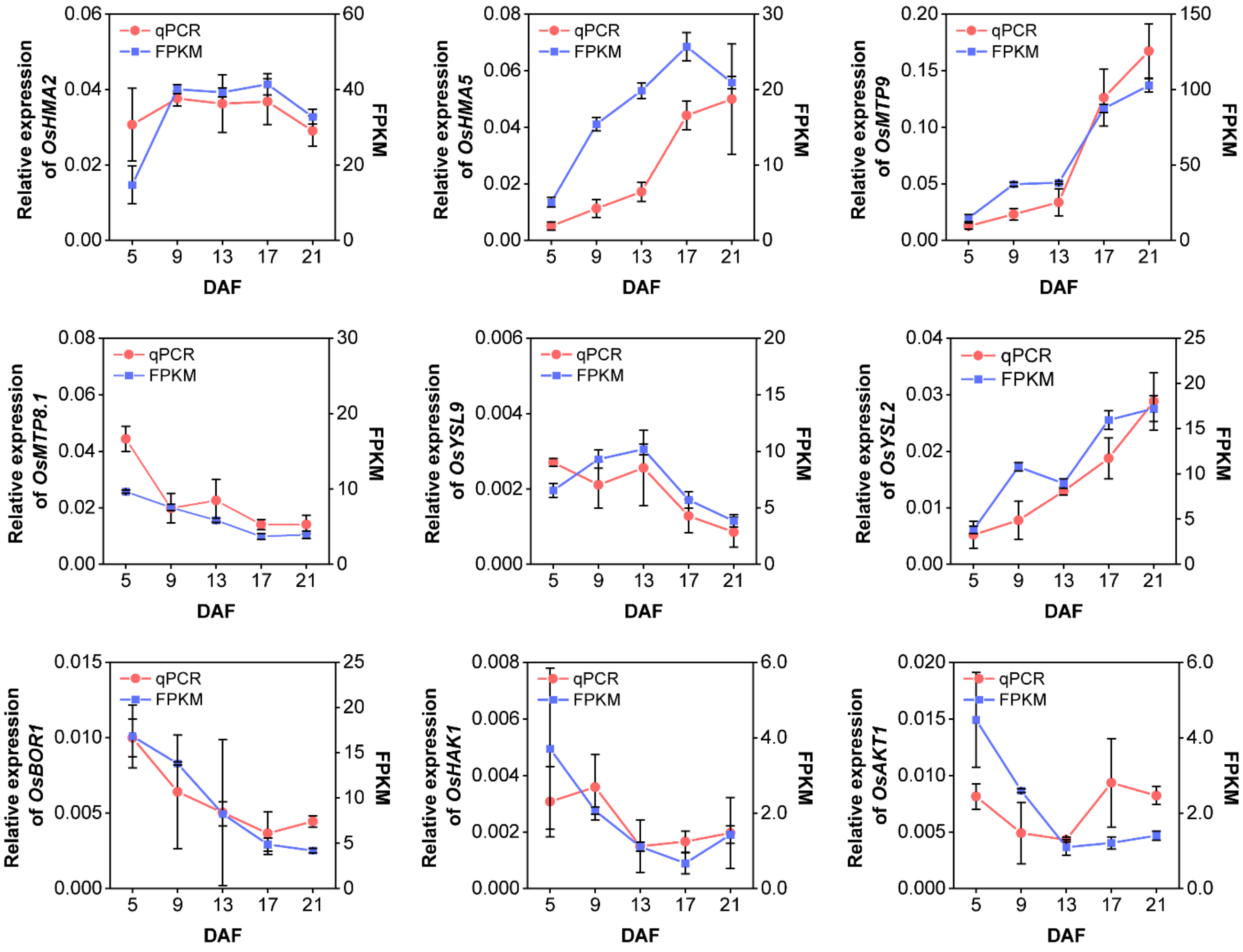

qPCR
FPKM
Relative expression of OsHMA2
Relative expression of OsHMA5
Relative expression of OsMTP9
Relative expression of OsMTP8.1
Relative expression of OsYSL9
Relative expression of OsYSL2
Relative expression of OsBOR1
Relative expression of OsHAK1
Relative expression of OsAKT1
DAF
5
9
13
17
21

B

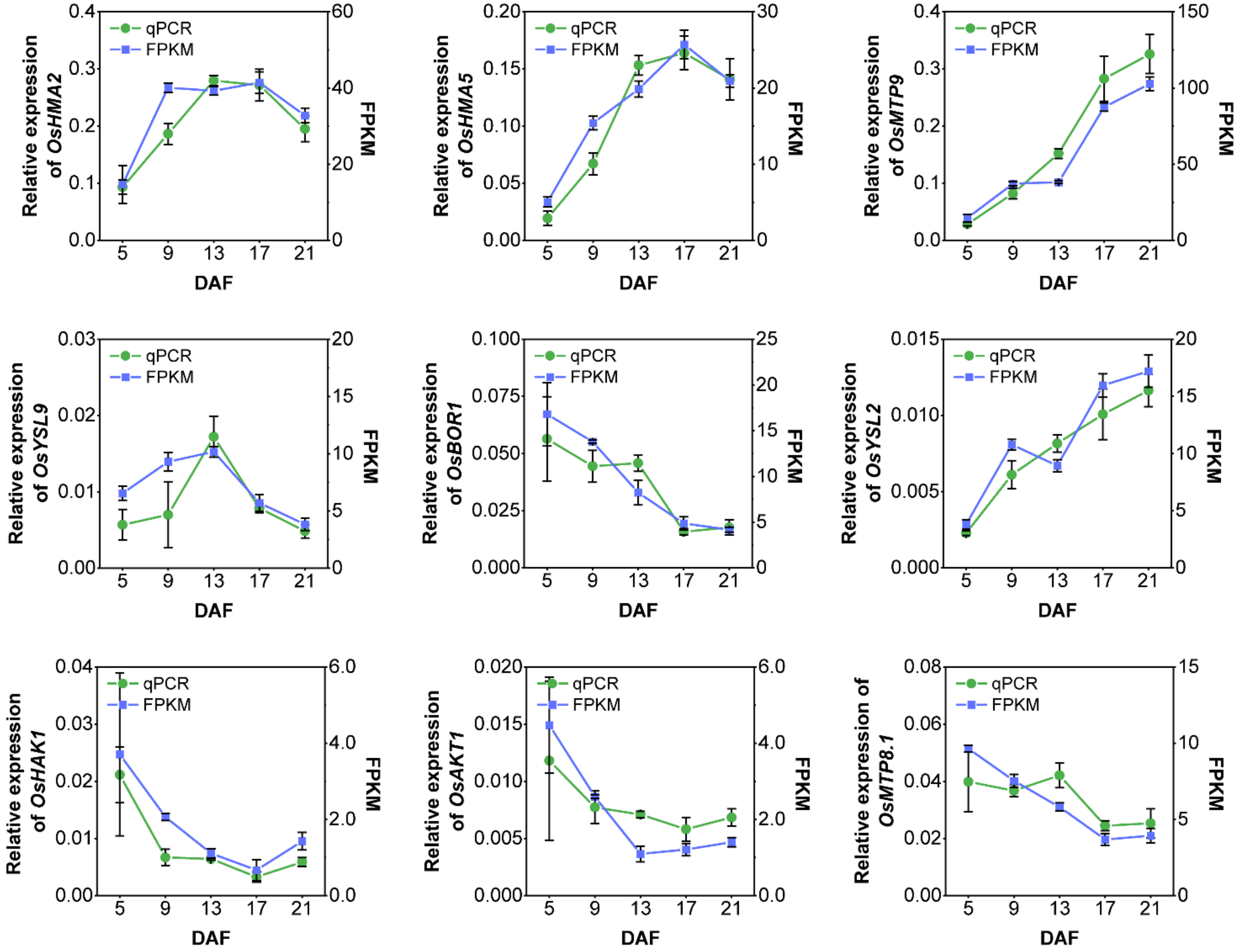

qPCR
FPKM
Relative expression of OsHMA2
Relative expression of OsHMA5
Relative expression of OsMTP9
Relative expression of OsYSL9
Relative expression of OsBOR1
Relative expression of OsYSL2
Relative expression of OsHAK1
Relative expression of OsAKT1
Relative expression of OsMTP8.1
FPKM
DAF
5
9
13
17
21

Figure S6. The expression levels of different ions transporter genes in developing grain (5-21 DAF). *Actin* (A) and *HistoneH3* (B) were used as an internal control for gene expression in of grain at the grain filling stage. Data at different developmental stages were obtained from three biological replicates, each biological replicate consisted of 30 grains collected from marked rachis. Error bars indicate standard deviations. DAF, days after fertilization.

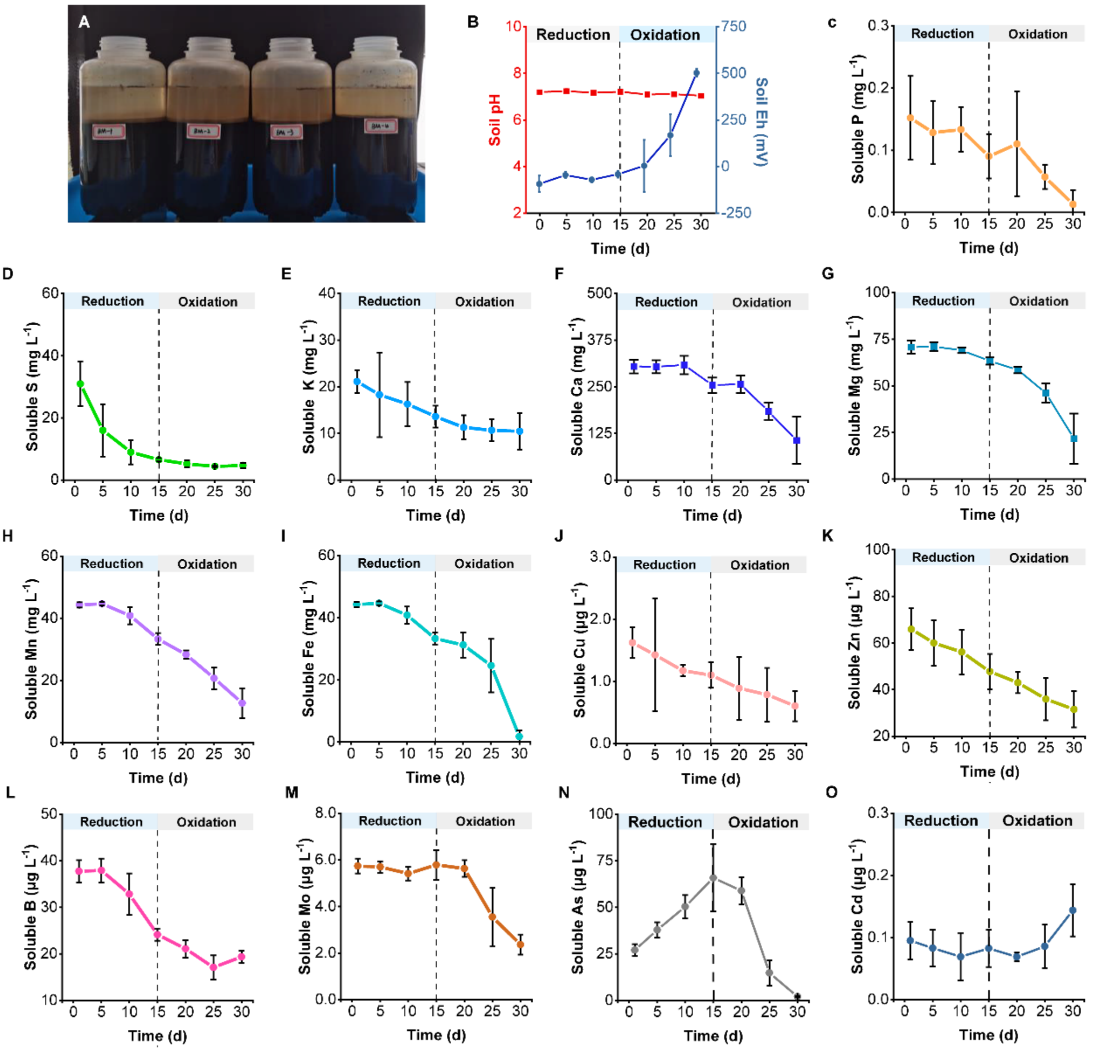

Figure S7. Temporal changes in soil soluble mineral element in the soils. (A) Microcosm experiment device. (B) Temporal changes in soil Eh and porewater pH. (C-O) Temporal changes in soil soluble mineral element in the soils as affected during 15 d reduction and subsequent 15 d oxidation during a microcosm experiment. Data are means ± SD (n = 4).

Table S1. Background properties of the soil from the experimental site.

Soil properties	Initial values
pH	7.15 ± 0.09
Total organic carbon	14.7 ± 0.4 g/kg
Available SO_4^{2-}	1.16 ± 0.04 mmol/kg
Total P	0.23 ± 0.04 g/kg
Total K	3.7 ± 0.4 g/kg
Total Ca	3000 ±240.3 mg/kg
Total Mg	3.15 ± 0.25 g/kg
Total Mn	667 ± 49.7 mg/kg
Total Fe	12700 ± 650 mg/kg
Total Cu	19.6 ± 1.7 mg/kg
Total Zn	43.6 ± 3.1 mg/kg
Total As	9.5 ± 1.2 mg/kg
Total Cd	0.06 ± 0.02 mg/kg

Table S2. Data filtering of reads.

Sample ID	Clean Reads No.	Clean Data (bp)	Clean Reads (%)	Clean Data (%)
DAF5-1	42504490	6349005953	98.7	98.7
DAF5-2	40225440	5998705150	98.4	98.4
DAF5-3	43076522	6430223131	98.5	98.5
DAF9-1	40149420	5994802089	98.5	98.5
DAF9-2	42392166	6329901375	98.3	98.3
DAF9-3	38245132	5711147751	98.5	98.5
DAF13-1	41459448	6185408921	98.9	98.9
DAF13-2	39295176	5869086889	99.1	99.1
DAF13-3	38590720	5765217947	99.0	99.0
DAF17-1	37346810	5579684827	99.1	99.1
DAF17-2	41168556	6147957289	99.2	99.2
DAF17-3	36960008	5516869626	98.9	98.9
DAF21-1	39520290	5895212753	98.8	98.8
DAF21-2	42374248	6320298172	98.7	98.7
DAF21-3	41254938	6155824784	98.8	98.8

Notes:

Sample ID: Sample name;

Clean Reads No: The number of reads for high-quality sequences;

Clean Reads (bp): The number of bases of the filtered high-quality sequence (bp);

Clean Reads (%): Percentage of high-quality reads in total reads sequenced (%);

Clean Data (%): Percentage of high-quality sequence bases to the total number of bases sequenced (%).

Table S3. Primer sets used in the present study.

	Forward	Reverse
OsHMA5	AAGGTGGAGAGTATAATGGTGAC	CCTTCCGGCCGACTGAAGTTC
OsHMA2	CATAGTGAAGCTGCCTGAGATC	GATCAAACGCATAGCAGCATCG
OsYSL9	TGACAAAAGCAAAGCAGCAC	CGCCTACAGGCTAAAACCAT
OsYSL2	TGCTGAGTTCGACATGGTATG	GGCCGGTCTATCCATTACCT
OsMTP8.1	AAGGAGGCACATGCTATTGG	ATGTTGTGCTCTGGCTTGTG
OsHAK1	GTTGATGATGCTGATGTTGGAAG	CCAACACTTTCAGCTGAAAC
OsAKT1	AGAGATCCTTGATTCACTGCC	TCTACTAACTCCACACTACCAG
OsBOR1	CACTAGAAGCCGTGGTGAAA	CAGGTAGTTGCATAGCTCAT
OsMTP9	AGGACCATTTCTTCGACGTG	TCCATCCACCATTTGTACCG
OsACTIN	GACTCTGGTGATGGTGTCAGC	GGCTGGAAGAGGACCTCAGG
HistoneH3	GGTCAACTTGTTGATTCCCCTCT	AACCGCAAAATCCAAAGAACG

Table S4. Programmer used for HNO_3-$HC1O_4$ digestions

Step	Ramp rate (°C h^{-1})	Dwell temp. (°C)	Dwell time (h)
1	60	60	3
2	120	100	1
3	120	120	1
4	50	190	2

Table S5. Linear regression equations of grain element concentration (y) in different parts of a panicle (x).

Element	Equation	R^2
N	y = 0.197 x – 0.853	0.748
P	y = 0.107 x – 0.466	0.645
S	y = -0.335 x + 1.11	0.853
K	y = 0.0886 x – 0.446	0.305
Mg	y = 0.113x – 0.453	0.213
Ca	y = 0.212 x – 0.975	0.795
Mn	y = -0.106 x + 0.249	0.166
Fe	y = 0.227 x – 1.04	0.887
Cu	y = 0.0676 x – 0.245	0.300
Zn	y = -0.116x + 0.498	0.473
B	y = 0.139 x – 0.282	0.154
Mo	y = 0.324 x – 1.64	0.863
As	y = -0.0214 x – 0.0888	0.0122
Cd	y = 0.0604 x – 0.384	0.0949

Table S6. Linear regression equations of grain element content (y) in different seed setting rates (x).

Element	Equation	R^2
N	y = -20.7 x + 262	0.664
P	y = -1.99 x + 75.6	0.141
S	y = -2.15 x + 23.8	0.225
K	y = -1.04 x + 52.5	0.0609
Mg	y = -0.665 x + 29.9	0.131
Ca	y = 0.0587 x + 2.50	0.00298
Mn	y = 0.0102 x + 0.640	0.0252
Fe	y = -0.102 x + 0.684	0.0350
Cu	y = -0.00665 x + 0.0713	0.455
Zn	y = -0.109 x + 1.01	0.725
B	y = -0.0102 x + 0.0653	0.314
Mo	y = -0.000465 x + 0.00682	0.173
As	y = 0.409 x + 2.40	0.135
Cd	y = -0.000370 x + 0.0370	0.000174
